# Unraveling the future productivity burden of cardiovascular disease in Qatar: Investigating the modifiable risk factors control in type 2 diabetes

**DOI:** 10.1016/j.ajpc.2025.100961

**Published:** 2025-03-11

**Authors:** Dina Abushanab, Daoud Al-Badriyeh, Danny Liew, Zanfina Ademi

**Affiliations:** a Health Economics and Policy Evaluation Research (HEPER), Centre for Medicine Use and Safety, Faculty of Pharmacy and Pharmaceutical Sciences, Monash University, Melbourne, Australia; b Department of Pharmacy, Hamad Medical Corporation, Doha, Qatar; c Clinical Pharmacy and Practice, College of Pharmacy, QU Health, Qatar University, Doha, Qatar; d The Adelaide Medical School, The University of Adelaide, Adelaide, Australia; e School of Public Health and Preventive Medicine, Monash University, Melbourne, Australia

**Keywords:** Cardiovascular disease, Diabetes, Productivity, Economics, Health, Projection

## Abstract

**Aims:**

Insufficient risk factor control can lead to a loss of millions of productivity-adjusted life years (PALYs). We aimed to assess the productivity burden of cardiovascular disease (CVD) in type 2 diabetes (T2D) and examine the potential advantages of enhancing the control of modifiable CVD risk factors in Qatar.

**Materials and methods:**

Models were developed to quantify the productivity burden, in terms of PALYs, of CVD in Qataris with T2D, aged 40–65 years, from 2024 to 2033. The financial value of PALYs was determined based on the gross domestic product (GDP) per full-time worker (i.e. US$80,573). The base-case model estimated the productivity burden of CVD, and interventional scenarios were simulated to assess potential gains resulting from improved control of modifiable risk factors, including reduced incidence of T2D, lower systolic blood pressure (SBP), decreased number of smokers, and reduced total cholesterol. All costs and outcomes were discounted at an annual rate of 3 %.

**Results:**

The base-case analysis projected that CVD in T2D would result in an estimated 2,096,536 PALYs (95 % confidence interval, 1,689,272–2,182,939), contributing US$225.46 (95 %CI, 1,689,272–2,182,939) billion to the country's GDP. However, implementing interventions to decrease the T2D incidence, lower SBP, reduce the number of smokers, and improve the total cholesterol could yield gains of 200,408, 198,173, 194,725, and 113,462 PALYs, respectively. These improvements would also lead to economic gains of US$20.01 billion, US$20.17 billion, US$19.78, and US$12.79 billion, respectively.

**Conclusions:**

Implementing interventions that prioritize risk factor control and prevention of CVD can help enhance overall productivity in the country.

## Graphical abstract

**Figure:**
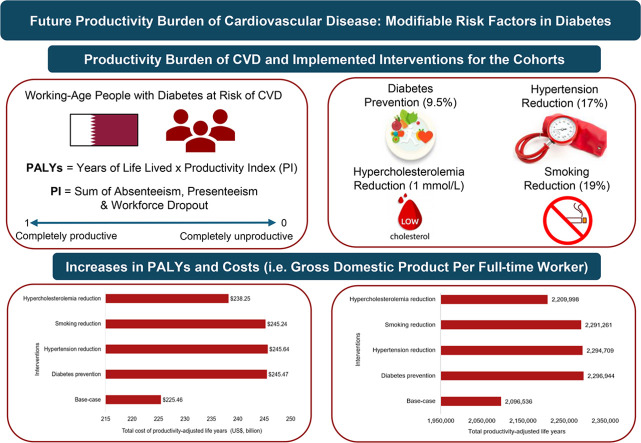

## 1 Introduction

Qatar confronts a significant prevalence of type 2 diabetes (T2D) [1]. In 2019, the prevalence of T2D among individuals aged 20–49 in Qatar varied from 17 % to 50 % [1]. Around 80 % of the population in the country has at least two risk factors for developing T2D [1,2]. In 2023, approximately 53.1 % of the population in Qatar was estimated to be obese, while 20.7 % were smokers [2]. By 2050, it is anticipated that nearly 32 % of Qatar's total healthcare expenditure will be allocated to addressing the demands of managing T2D [2].

Individuals with T2D are at increased risk of developing cardiovascular disease (CVD) [3], with over 190,000 expected CVD events in the next decade, costing more than US$20 billion in direct and productivity-related losses [4].

Addressing CVD in people with T2D is a crucial priority for policymakers in Qatar, aligning with the National Health Vision 2030 [5]. While current public health efforts focus on behavioral interventions to reduce CVD risks associated with T2D, these strategies have not been successful in alleviating the burden of CVD, especially among the working-age population [6]. To tackle the pressing issue of CVD in T2D in Qatar, it is important that the World Health Organization (WHO) has established a set of highly cost-effective policy options known as “best buys” [7]. These strategies focus on mitigating behavioral and metabolic risk factors associated with non-communicable diseases (NCDs), including clinical interventions for prevention and treatment. However, their implementation remains inadequate, often due to a lack of awareness regarding the hidden economic costs of NCDs, which can overshadow the financial rationale for action [7]. In Qatar, the Ministry of Public Health has prioritized quantifying the costs of interventions and their potential return on investment. By developing investment cases, the ministry aims to create a compelling economic argument for adopting recommended interventions to prevent and control NCDs, aligning with National Health Vision 2030 [8]. Despite these efforts, significant challenges persist. Limited public awareness regarding the risk factors and complications associated with diabetes and CVD hampers effective prevention strategies [8,9]. The healthcare system in Qatar faces fragmentation, complicating the delivery of integrated care essential for managing these chronic diseases [10,11]. There are also gaps in epidemiological data related to diabetes and CVD, complicating effective planning and intervention [7]. Socioeconomic factors such as rapid urbanization, lifestyle changes, rising obesity rates, physical inactivity, and unhealthy diets further exacerbate these issues [8,9]. Cultural beliefs and practices also influence health-seeking behaviors and adherence to preventive measures and treatments [7]. These factors affect people health outcomes and have significant implications for overall productivity within the population. Therefore, a comprehensive and multi-faceted approach is required to combat diabetes and CVD effectively in Qatar, ensuring that all population groups understand their rights to health and well-being.

An increasingly recognized dimension of burden is the impact of health conditions on work productivity at a population level [12]. To investigate the productivity burden, a novel measure known as the productivity-adjusted life-year (PALY) has been devised [12], which considers the loss of productivity attributed to diseases. While a prior study conducted by our group in Australia has examined CVD burden in terms of PALYs in T2D [1], the findings of that study do not accurately represent the Qatari context. Furthermore, no study has utilized dynamic modeling to estimate the burden of CVD in T2D in terms of PALYs in the Middle East and North Africa (MENA). Therefore, this study aimed to employ a 10-year dynamic modeling approach to examine the epidemiological impact of CVD in T2D in terms of PALYs in Qatar and evaluate the effects of four interventions from 2024 to 2033.

## 2 Materials and methods

### 2.1 Model overview

A validated model of CVD in individuals with T2D was adapted [13] to quantify the epidemic of CVD in terms of years of life lived and PALYs among the working-age population with T2D in Qatar. Age- and sex-specific models were developed for individuals aged 40–65 from 2024 to 2033, using yearly cycles. The model was dynamic, incorporating the entry and exit of individuals over the ten-year follow up due to factors such as mortality, immigration, emigration, and new cases of T2D. The assumed retirement age in Qatar is 65 years, as indicated by the Organization for Economic Co-operation and Development [14]. Consequently, individuals were included in the model starting at age 40 and then exited at age 66, reflecting the working-age demographic. The selection of the starting age of 40 was based on prior research that focused on the health and the direct and indirect economic burden of CVD in individuals aged 40 to 79 with T2D in Qatar [15], as this age group experiences a significant economic impact from T2D. Data also suggest that T2D affects around 10 % of individuals aged 18 to 64 years [16].

**Fig. 1:**
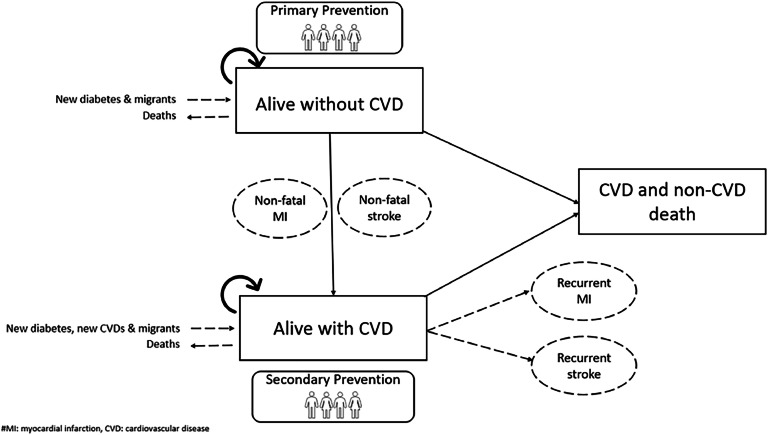
A two-stage dynamic multistate model in individuals with type 2 diabetes with and without cardiovascular disease over a 10-year period.

In this study, two-stage dynamic multistate Markov models were developed using Microsoft Excel to estimate the burden of CVD among individuals with T2D in terms of PALYs (Fig. 1). The models tracked individuals over ten years, following their status until death or reaching 66 years of age. They accounted for both incident and recurrent non-fatal cardiovascular events, such as myocardial infarction (MI) and stroke, as well as mortality from both cardiovascular and non-cardiovascular causes among individuals with and without established CVD. Individuals aged 40 to 66 with T2D and no prior CVD began in the ‘Alive with T2D without established CVD’ state, where they could experience non-fatal or fatal cardiovascular events, leading to transitions to either the ‘Alive with T2D with established CVD’ state or the ‘Dead’ state. Half-cycle corrections were applied between years to enhance accuracy.

The base-case analysis was designed to quantify the burden of CVD in T2D in terms of PALYs, using real-world data from the Primary HealthCare Corporation (PHCC) for 2016 to 2021, which serves as Qatar's principal provider of primary care across 31 clinics [17]. A detailed description of the model is available in our previously published work [15]. Briefly, in the first stage of the model, the risk of incident CVD among individuals with T2D was calculated using the 2013 Pooled Cohort Equations for Atherosclerotic Cardiovascular Disease (PCE-ASCVD), which estimates the ten-year absolute rate of incident CVD based on various risk factors such as sex, age, smoking status, systolic blood pressure (SBP, diabetes status, hypertension treatment status, total cholesterol, and high-density lipoprotein cholesterol levels (HDL)). The model was parameterized using risk estimates from the Qatari PHCC. Age- and sex-specific average ten-year risk scores were calculated and converted into annual probabilities. These annual event rates were plotted against the midpoints of each ten-year age group, with polynomial functions applied to model values for each age year. In the second stage, due to the lack of contemporary data on recurrent CVD events among individuals with T2D in Qatar, estimates from the global Reduction of Atherothrombosis for Continued Health (REACH) registry were utilized. This registry followed 30,043 individuals over four years. As the REACH registry reports overall cardiovascular risk rates, these were adjusted to reflect single-age event probabilities, assuming that age impacts non-fatal and fatal CVD equivalently. Age- and sex-specific non-CVD mortality rates for the general Qatari population were sourced from the Ministry of Development Planning and Statistics. Supplementary Tables 1 to 5 provide comprehensive details regarding the inputs and data sources utilized in the simulation models.

To understand the potential health and productivity gains resulting from enhanced control of risk factors, we implemented four hypothetical cohort interventions. The model assumed that the improvements in risk factor control in these four scenarios would be sustained throughout the entire duration of the analysis.

The development of these scenarios was based on evidence derived from experimental designs or observational studies in the global literature, which indicated the potential benefits of these interventions in reducing CVD risk among individuals with T2D. Moreover, the feasibility and relevance of these scenarios were assessed to ensure their appropriateness within Qatar's cultural and socio-economic context. Please refer to Supplementary Table 6 for a detailed description of the interventional cohorts.

### 2.2 Outcomes

The primary outcomes were to quantify the magnitude of the CVD burden among individuals with T2D in terms of PALYs experienced over 10 years from 2024 to 2033, and to estimate the potential productivity benefits that could be achieved by enhancing the control of CVD risk factors over the same period. The analysis accounted for differences in the years of life lived and the PALYs gained through improved CVD risk factor control based on the evidence-based hypothetical scenarios. Additionally, the economic costs of PALYs were considered in relation to the country's gross domestic product (GDP). All findings were presented as discounted values, using an annual discount rate of 3 % [18].

### 2.3 Productivity indices

To determine the productivity indices for all the generated scenarios, we calculated the total number of working days missed in a year, considering absenteeism, presenteeism, and workforce dropout. This calculation was performed as a percentage of the maximum number of working days in a year for individuals aged 40 to 66 years. PALYs were then estimated by multiplying the years of life lived by the corresponding productivity indices. The productivity index ranges from 0 (indicating no productivity) to 1 (indicating full productivity) and takes into consideration the reduced work productivity resulting from absenteeism, workforce dropouts, and presenteeism [12].

### 2.4 Absenteeism

A study by Sørensen and Jon Ploug [19] found that individuals with T2D lost an average of 92 workdays in the first year due to MI, decreasing to 56 days in subsequent years. For stroke, the average was 125 days initially, dropping to 71 days later. In contrast, those without MI or stroke averaged 38 absent days each year.

### 2.5 Presenteeism

Data from Goetzel et al. [20] indicated that individuals with T2D and CVD reported an average of 7 unproductive workdays annually. The study combined estimates for unproductive time from both conditions.

### 2.6 Workforce dropout

The model estimated that 9 % of individuals do not return to work after an MI [21], increasing to 38 % after a stroke [22]. The average return time post-MI was 60.34 days [21], and 61 days post-stroke [22]. Due to a lack of specific data, early retirement rates for individuals with T2D were assumed to match general trends in the Qatari population.

For further details regarding the data on productivity indices, please refer to Supplementary Table 5.

The labor force participation was incorporated into the models to adjust the PALYs. This included employed, unemployed, and those not in the labor force. The productivity index assumes that a full-time worker has 240 working days per year, based on a 5-day, 40-hour workweek and 4 weeks of annual leave. The productivity index was calculated by subtracting absence and unproductive days from 240 days, before dividing by 240 days. PALYs were then computed by multiplying years of life by the productivity indices, further adjusted for workforce participation across age and sex groups.

To determine the economic value of PALYs, the GDP per full-time worker was calculated by multiplying PALYs by the GDP per worker for each year. Future GDP per hour was projected using 4.2 % annual growth, based on 2022 Qatar GDP per employed person of US$80,573 (using an exchange rate of US$1 = QAR3.65) [23]. GDP per full-time worker was then derived by multiplying GDP by annual working hours of 1920 (40 h/week x 48 weeks) [24]. The proportionate number of equivalent full-time (EFT) workers by age and sex was estimated using labor force participation and average hours worked data, assuming 40 h per week for full-time.

### 2.7 Sensitivity analyses

To further examine the relevance of uncertainty surrounding the key inputs in our base-case model, we conducted probabilistic sensitivity analyses (PSA). This involved adjusting the parameter values to the lower and upper bounds of their confidence intervals (CIs) through a Monte Carlo simulation, based on 10,000 iterations. When CIs were unavailable for specific parameters, we assumed a 10 % standard error around the mean. We assigned uncertainty ranges to the proportions of CVD events for individuals with T2D, both with and without prior CVD, as well as the transition probabilities for recurrent events in T2D and the productivity indices. Additionally, we considered *a* ± 25 % uncertainty range for Qatar's GDP and annual growth rate using a triangular distribution. The PSA was conducted using the @Risk-7.5® software Palisade Corporation, NY, US. A comprehensive breakdown of the key input parameters and their corresponding probability distributions can be seen in Supplementary Table 7.

We also conducted multiple scenario analyses to explore the impact of variations in key input data on the results in terms of years of life lived, PALYs, and associated costs. One scenario involved using the upper bound estimate for the number of absent days, as reported by Sørensen and Jon Ploug [19]. This allowed us to assess the potential impact of higher levels of absenteeism on the results. Another scenario involved assuming a constant GDP value over time. By considering the GDP constant, we aimed to evaluate the effect of removing the economic growth factor from our calculations. Furthermore, we replaced Qatar's GDP and annual growth rates with those of the Middle East, using an average GDP per capita of US$13,960 and an annual growth rate of 3.8 %, which provided insights into accounting for potential regional disparities [25]. Moreover, we explored the impact of changing the annual discount rate to 1 % and 5 % in additional scenarios.

### 2.8 Model validation and calibration

To ensure the accuracy and reliability of the model, we implemented a rigorous validation process. This involved carefully assessing the consistency of our assumptions and inputs used in the model with the latest research findings. We also examined the robustness of our results by varying key parameters. The model's validity was evaluated through the application of validation tools such as the Assessment of the Validation Status of Health-Economic decision models (AdViSHE) and TECHnical VERification (TECH-VER) [26,27]. In addition to these validation tools, independent checks were conducted within the Excel spreadsheet to identify any potential modeling errors. This further supported the face validity of our modeling approach and the reliability of our data sources. We also calibrated the dynamic model using incidence rates of fatal and non-fatal cardiovascular events in the Qatari population [15]. The calibration process is essential for ensuring that our model accurately reflects local healthcare dynamics and epidemiological trends.

## 3 Results

### 3.1 Years of life lived

In the base-case model from 2024 to 2033, our model projected a total of 4822,224 (95 %CI, 4.773,109–5010,628) years of life lived in the population with T2D and both first and recurrent CVD. Males and females were predicted to accrue 3818,836 and 1003,387 years of life lived. When we considered a 9.5 % reduction in the incidence of T2D, our model projected a gain of 4558,633 (95 %) years of life lived in total cohort. Implementing a 17 % reduction in SBP, a 19 % reduction in the number of smokers, and a 1 mmol/L reduction in total cholesterol resulted in additional gains of 455,817 (9 %), 447,885 (9 %), and 227,097 (5 %) years of life lived, respectively.

Table 1 presents a detailed forecast of the years of life lived for all cohorts within the Qatari population over the coming decade.

**Table 1:** The cumulative number of years lived by individuals in a Qatari cohort with type 2 diabetes who also have first and recurrent cardiovascular disease.

**Year**	**2024**	**2025**	**2026**	**2027**	**2028**	**2029**	**2030**	**2031**	**2032**	**2033**	**Sum (2024–33)**
**Base-case**	234,040	455,082	475,908	495,066	512,062	524,742	533,385	540,008	544,310	507,619	4822,224
**Cohort 1 (scenario 1)**	875,397	911,464	944,834	974,670	998,271	1013,964	1024,076	1029,997	1065,628	542,556	9380,856
**Difference (cohorts base-case and 1)**	641,357	456,382	468,925	479,603	486,209	489,221	490,691	489,989	521,319	34,937	4558,633 (95 %)^#^
**Cohort 2 (scenario 2)**	435,493	463,153	489,425	513,909	536,057	553,647	566,959	578,013	586,497	554,887	5278,040
**Difference (cohorts base-case and 2)**	201,453	8071	13,516	18,843	23,995	28,905	33,574	38,004	42,187	47,268	455,817 (9 %)^#^
**Cohort 3 (scenario 3)**	435,618	463,082	489,152	513,430	535,370	552,760	565,877	576,739	585,038	553,043	5270,109
**Difference (cohorts base-case and 3)**	201,578	8000	13,244	18,364	23,309	28,018	32,492	36,731	40,728	45,423	447,885 (9 %)^#^
**Cohort 4 (scenario 4)**	229,503	456,025	482,270	506,908	529,133	546,894	560,390	571,609	580,269	586,319	5049,321
**Difference (cohorts base-case and 4)**	(4537)	943	6361	11,842	17,071	22,152	27,005	31,600	35,959	78,700	227,097 (5 %)^#^

*Base-case: original model with original estimates from the Primary Healthcare Corporation without any adjustment in risk factors.

^#^Percentage of the increase in the number of years lived with interventions relative to the base case.

Cohort 1 (scenario 1): the model assumed a 9.5 % reduction in the incidence of type 2 diabetes.

Cohort 2 (scenario 2): the model assumed a 17 % reduction in hypertension.

Cohort 3 (scenario 3): the model assumed a 19 % reduction in the number of smokers.

Cohort 4 (scenario 4): the model assumed a 39 mg/dl reduction in hypercholesterolemia.

### 3.2 PALYs and GDP

In the base-case model, our estimates indicate a total of 2096,536 (95 %CI, 1689,272–2182,939) PALYs, which contributed approximately US$225.46 (95 %CI, 223.09–229.57) billion to the country's GDP. The estimates for PALYs in males and females were 1660,298 (equivalent to US$178.47 billion) and 436,238 (equivalent to US$46.99 billion) respectively.

**Fig. 2:**
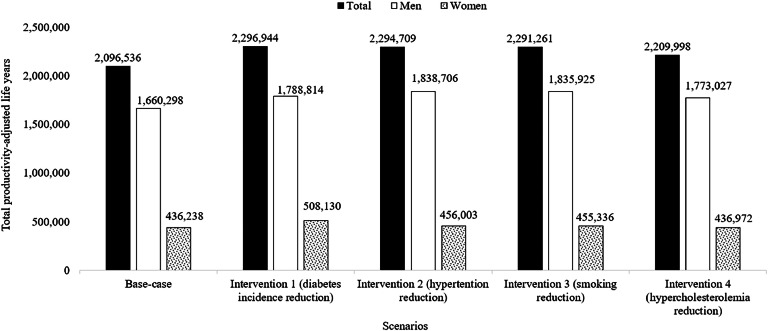
The productivity-adjusted life years (PALYs) in all cohorts of the population with diabetes, including first and recurrent cardiovascular disease events over a 10-year period.

**Fig. 3:**
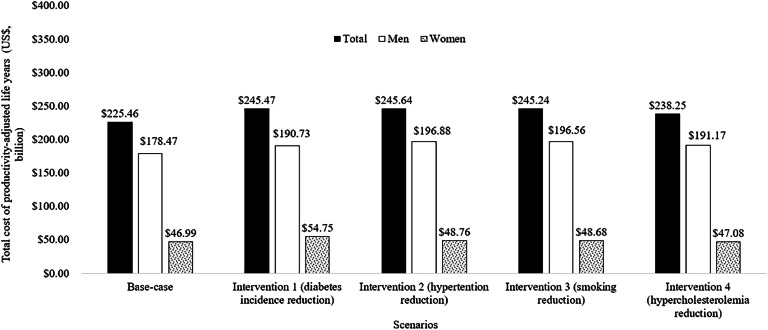
The cost of productivity-adjusted life years (PALYs) in all cohorts of the population with diabetes, including first and recurrent cardiovascular disease events over a 10-year period.

By reducing the incidence of T2D by 9.5 %, our model projected gains of 200,408 (10 %) PALYs, accompanied by economic gains of approximately US$20.01 (9 %) billion. Furthermore, if there were a 17 % reduction in SBP, a 19 % reduction in smoking rates, and a 39 mg/dl reduction in total cholesterol, we would observe gains of 198,173 (9 %), 194,725 (9 %), and 113,462 (5 %) PALYs, respectively. These reductions would also result in economic gains of approximately US$20.17 (9 %) billion, US$19.78 (9 %) billion, and US$12.79 (6 %) billion, respectively (Figs 2 and 3).

A detailed breakdown of the PALYs and broader economic costs for the Qatari population over the next decade, in all cohort intervention scenarios, can be seen in Table 2, Table 3.

**Table 2:** The cumulative productivity-adjusted life-years by individuals in a Qatari cohort with type 2 diabetes who also have first and recurrent cardiovascular disease.

**Year**	**2024**	**2025**	**2026**	**2027**	**2028**	**2029**	**2030**	**2031**	**2032**	**2033**	**Sum (2024–33)**
**Base-case**	101,753	197,854	206,909	215,238	222,627	228,140	231,897	234,777	236,647	220,695	2096,536
**Cohort 1 (scenario 1)**	198,475	207,698	216,386	224,364	231,415	236,605	240,125	242,775	258,456	240,646	2296,944
**Difference (cohorts base-case and 1)**	96,722	9844	9478	9126	8789	8465	8227	7998	21,809	19,950	200,408 (10 %)^#^
**Cohort 2 (scenario 2)**	189,337	201,363	212,785	223,430	233,059	240,707	246,494	251,300	254,989	241,246	2294,709
**Difference (cohorts base-case and 2)**	87,585	3509	5876	8192	10,432	12,567	14,597	16,523	18,342	20,550	198,173 (9 %)^#^
**Cohort 3 (scenario 3)**	189,392	201,332	212,666	223,222	232,761	240,321	246,024	250,746	254,354	240,444	2291,261
**Difference (cohorts base-case and 3)**	87,639	3478	5758	7984	10,134	12,181	14,126	15,969	17,707	19,749	194,725 (9 %)^#^
**Cohort 4 (scenario 4)**	103,014	201,567	213,003	223,694	233,300	240,956	246,743	251,541	255,216	240,963	2209,998
**Difference (cohorts base-case and 4)**	1262	3713	6094	8456	10,673	12,816	14,846	16,764	18,569	20,268	113,462 (5 %)^#^

*Base-case: original model with original estimates from the Primary Healthcare Corporation without any adjustment in risk factors.

^#^Percentage of the increase in the productivity-adjusted life-years with interventions relative to the base case.

Cohort 1 (scenario 1): the model assumed a 9.5 % reduction in the incidence of type 2 diabetes.

Cohort 2 (scenario 2): the model assumed a 17 % reduction in hypertension.

Cohort 3 (scenario 3): the model assumed a 19 % reduction in the number of smokers.

Cohort 4 (scenario 4): the model assumed a 39 mg/dl reduction in hypercholesterolemia.

**Table 3:** The cumulative cost of productivity-adjusted life-years in US$ in a Qatari cohort with type 2 diabetes who also have first and recurrent cardiovascular disease.

**Year**	**2024**	**2025**	**2026**	**2027**	**2028**	**2029**	**2030**	**2031**	**2032**	**2033**	**Sum (2024–33)**
**Base-case**	$8901,603,275	$18,035,784,549	$19,653,349,588	$21,303,170,266	$22,959,954,195	$24,516,726,368	$25,967,193,712	$27,393,791,776	$28,771,699,080	$27,959,240,140	$225,462,512,948
**Cohort 1 (scenario 1)**	$17,363,134,575	$18,933,129,620	$20,553,595,253	$22,206,435,400	$23,866,349,444	$25,426,423,557	$26,888,438,834	$28,326,986,567	$31,423,217,759	$30,486,694,364	$245,474,405,373
**Difference (cohorts base-case and 1)**	$8461,531,301	$897,345,072	$900,245,665	$903,265,134	$906,395,250	$909,697,189	$921,245,123	$933,194,791	$2651,518,679	$2527,454,224	$20,011,892,425 (9 %)^#^
**Cohort 2 (scenario 2)**	$16,563,776,238	$18,355,651,409	$20,211,518,759	$22,113,993,666	$24,035,847,570	$25,867,208,400	$27,601,699,223	$29,321,691,943	$31,001,687,386	$30,562,712,375	$245,635,786,970
**Difference (cohorts base-case and 2)**	$7662,172,963	$319,866,860	$558,169,171	$810,823,400	$1075,893,376	$1350,482,032	$1634,505,511	$1927,900,167	$2229,988,306	$2603,472,236	$20,173,274,022 (9 %)^#^
**Cohort 3 (scenario 3)**	$16,568,531,899	$18,352,826,150	$20,200,270,505	$22,093,369,078	$24,005,068,831	$25,825,745,631	$27,549,006,419	$29,257,100,681	$30,924,542,769	$30,461,121,821	$245,237,583,784
**Difference (cohorts base-case and 3)**	$7666,928,624	$317,041,601	$546,920,917	$790,198,812	$1045,114,637	$1309,019,263	$1581,812,707	$1863,308,905	$2152,843,689	$2501,881,681	$19,775,070,836 (9 %)^#^
**Cohort 4 (scenario 4)**	$9011,996,478	$18,374,227,653	$20,232,232,508	$22,140,120,464	$24,060,697,768	$25,894,020,216	$27,629,599,548	$29,349,869,326	$31,029,342,973	$30,526,935,049	$238,249,041,987
**Difference (cohorts base-case and 4)**	$110,393,203	$338,443,104	$578,882,920	$836,950,198	$1100,743,573	$1377,293,848	$1662,405,836	$1956,077,550	$2257,643,893	$2567,694,910	$12,786,529,040 (6 %)^#^

*Base-case: original model with original estimates from the Primary Healthcare Corporation without any adjustment in risk factors.

^#^Percentage of the increase in the cost of productivity-adjusted life-years with interventions relative to the base case.

Cohort 1 (scenario 1): the model assumed a 9.5 % reduction in the incidence of type 2 diabetes.

Cohort 2 (scenario 2): the model assumed a 17 % reduction in hypertension.

Cohort 3 (scenario 3): the model assumed a 19 % reduction in the number of smokers.

Cohort 4 (scenario 4): the model assumed a 39 mg/dl reduction in hypercholesterolemia.

Additionally, a granular analysis focusing on individuals with first CVD, recurrent CVD, and the total population (first and recurrent), stratified by sex, is found in Supplementary Tables 8–16.

### 3.3 Sensitivity analysis

In comparison to the base-case scenario, increasing the number of absent days led to a reduction of 531,621 PALYs and a decrease in GDP by US$57.22 billion (equivalent to 25 %). When assuming no economic growth over time, the GDP decreased by US$56.54 billion compared to the base case (25 %). By replacing the average GDP per capita and annual growth rate with those of the Middle East, we observed a significant reduction in the cost of PALYs by US$187.46 billion (83 %). On the contrary, adjusting the annual discount rate to 1 % and 5 % resulted in a GDP increase of US$24.39 billion (10 %) and a decrease of US$21.05 billion (9 %) compared to the base case. The findings of the PSA are available in Table 4 and the outcomes of the scenario analyses can be found in Table 5.

**Table 4:** Outcomes of probabilistic sensitivity analyses for the base-case model in total population over 10 years.

**Outcome**	**Total, Mean (LL and UL of uncertainty range)**
**Years of life lived**	4822,224 (4631,001–4996,729)
**Productivity-adjusted life-year**	2096,536 (1975,942–2103,917)
**Cost of productivity-adjusted life-year (US$)**	225,462,512,948 (224,673,974–225,986,721)

LL: lower limit, UL: upper limit.

**Table 5:** Scenario analyses in total population over 10 years.

**Scenario**	**Years of life lived**	**Productivity-adjusted life-years**	**Cost of productivity-adjusted life-years (US$)**
**Base-case**			
	4822,224	2096,536	225,462,512,948
**Using the upper bound estimate for absent days**			
	4822,224	1564,915	168,236,308,472
**Assuming a constant GDP**^#^**over time**			
	4822,224	2096,536	168,923,329,493
**Reducing the annual discount rate to 1 %**			
	5312,562	2309,718	249,850,554,733
**Increasing the annual discount rate to 5 %**			
	4397,385	1911,831	204,408,840,016
**Replacing the GDP**^#^**and annual growth of Qatar with the averages for the Middle East**			
	4822,224	2096,536	38,003,502,327

*Base-case: original model with original estimates from the Primary Healthcare Corporation without any adjustment in risk factors.

^#^GDP: gross domestic product.

### 3.4 Model validation

The calibration ratios indicated that our model may overestimate the health and economic impact of CVD among individuals with T2D in Qatar. The detailed results of calibration were previously published [15]. External validation suggests that our model may overestimate the health and economic burden of CVD in the with and without CVD populations with T2D in Qatar. The validation of model checklists is presented in Supplementary Table 17.

## 4 Discussion

This study is the first of its kind in the MENA region, projecting the burden of CVD in terms of PALYs among people with T2D in Qatar over the next decade. The baseline projections from the model indicate that without any changes to current health policies, expenditures, prevention efforts, or early disease management, the burden of CVD among people aged 40–65 years with T2D in Qatar would be 2096,536 PALYs. In addition, this would contribute an estimated US$225.46 billion to the country's GDP. However, our analysis also examined interventions that simulated targeted reductions in key risk factors. The findings revealed that decreases in T2D incidence, hypertension, smoking prevalence, and hypercholesterolemia, could lead to significant gains of 200,408 (10 %), 198,173 (9 %), 194,725 (9 %), and 113,462 (5 %) PALYs, respectively. These improvements in risk factors would not only enhance health outcomes, but also generate substantial economic benefits, ranging from US$12.69 (6 %) to US$20.17 billion (9 %). It is important to note that in the diabetes incidence reduction intervention, the years of life lived in 2033 decrease compared to previous trends, which could occur despite diabetes prevention interventions due to factors such as population aging, saturation of benefits, and the development of complications from T2D. These findings underscore the urgent need to strengthen primary prevention strategies at the population level to address the looming burden of CVD among individuals with T2D in Qatar. Further, our study highlights the importance of local economic contexts in health economic modeling. By incorporating region-specific GDP values, we demonstrated a dramatic decrease in estimated costs associated with PALYs, from approximately US$225.46 billion to around US$38 billion. This emphasizes the necessity of tailoring health economic models to reflect local realities, particularly in regions like the MENA, where socioeconomic factors significantly impact health outcomes.

The calibration process was conducted to accurately reflect local healthcare dynamics and epidemiological trends. This approach was important for aligning our model with the realities of Qatar's healthcare environment, enabling more relevant and applicable predictions. Our calibration findings indicated the model overestimated non-fatal MI, non-fatal stroke, and CVD death in people with T2D at risk and with CVD. These overestimated ratios suggest that while our model is rooted in Qatari data, it may present an upward bias in its estimates of cardiovascular events. This could potentially lead to inflated projections regarding the burden of CVD in the population, which is an important consideration for public health planning and resource allocation. For example, if the model predicts mortality or CVD event rates that exceed observed rates, it may create an inflated perception of the CVD burden within the population. This could lead to an erroneous emphasis on CVD prevention strategies. Also, clinicians might adopt more aggressive treatment protocols based on inflated event risks, potentially resulting in unnecessary interventions. Therefore, future research should focus on longitudinal studies and collaborations with local health institutions to gather more precise data, which would help further refine the model and improve its accuracy and applicability.

Our analysis reveals a concerning trend in Qatar, projecting a significant rise in the costs associated with PALYs due to CVD among people with T2D over the next decade, which suggests a growing pressure on the country's healthcare system in the coming years. However, CVD is a largely preventable condition and, hence, this study should serve as a crucial resource for decision-makers and policymakers to prioritize and implement robust prevention strategies, particularly regular health screenings for risk factors such as high blood pressure and high cholesterol. This is especially relevant considering that the proportion of potential PALYs among the at-risk population exceeded that of people with existing CVD in the current study.

The findings are not only significant for Qatar but also relevant for the broader GCC region, which shares similar demographic and socioeconomic profiles [28,29]. The MENA region, including the GCC, faces a heightened risk of MI and is projected to see a 140.9 % rise in CVD prevalence due to increasing rates of ischemic heart disease and obesity [29]. These trends are likely mirrored in the Gulf. While GCC countries are investing in healthcare infrastructure for universal health coverage [29,30], rapid economic growth and demographic changes pose challenges, particularly with rising NCDs like CVD. Additionally, the projected productivity burden highlights the socioeconomic impact of CVD, especially for nations with a large expatriate workforce [30,31]. Addressing indirect costs related to morbidity and mortality can inform policies aimed at improving workforce participation and promoting workplace wellness.

Furthermore, the study findings reveal a notable sex disparity, with projected PALYs and associated costs being approximately three times higher in men than in women. This disparity is likely attributable to the demographic composition of Qatar's population, where migrant male workers comprise over 70 % of the total population [32].

Although comparisons to international studies provide some context [[33], [34], [35], [36], [37], [38]], they may not be directly applicable to the Qatari setting. For example, a recent study conducted in Australia by Abushanab et al. [13] projected 1.21 million PALYs lost due to CVD in individuals with T2D aged 40–69, which is lower than the figures reported for Qatar. The economic losses projected in the Australian study (AU$258.93 billion or US$168.30 billion), are also below our estimates. The Australian study also demonstrated that addressing key risk factors, such as reducing hypertension, lowering the number of smokers, decreasing hypercholesterolemia, and diminishing the incidence of T2D, could potentially yield gains of 7889, 28,971, 7117, and 320,124 PALYs, respectively. These improvements correspond to economic benefits of AU$1.72 billion (US$1.12 billion), AU$6.21 billion (US$4.04 billion), AU$1.55 billion (US$1.01 billion), and AU$68.34 billion (US$44.43 billion), all of which are also lower than the economic gains projected in our present study. This discrepancy is not surprising, given that both diabetes and CVD prevalence rates in Qatar are significantly higher than those in Australia [39]. Furthermore, in contrast to the current study, which analyzed data from over 48,000 individuals to estimate CVD risk for the population with T2D but without a history of CVD, the Australian study was based on a much smaller cohort of only 313 participants [40]. This larger sample size in our research enhances the reliability of the first-ever estimates generated from the PCE-ASCVD equation within the MENA region. It is also important to note that Qatar's GDP is higher than that of Australia [23], which may further influence the economic implications of these findings.

Addressing CVD in individuals with T2D is a critical priority for Qatar's policymakers, aligning with the National Health Vision 2030 [6,[41], [42]]. Despite campaigns aimed at reducing tobacco use and promoting physical activity, smoking rates remain high among specific demographics, indicating that current measures may not effectively reach all population segments. Similarly, the rising obesity rates suggest that existing interventions lack the comprehensive strategies required for sustained behavioral change. To enhance primary prevention strategies, it is essential to develop a cohesive national framework that integrates various approaches. Engaging multiple stakeholders, including healthcare providers, community organizations, and the private sector, can create a supportive environment for healthy lifestyle choices. Moreover, addressing socioeconomic determinants, such as income and access to healthcare, is important for improving health outcomes. In addition, technological innovations, including mobile health applications and telemedicine, can enhance monitoring and encourage positive lifestyle changes. Furthermore, policymakers should consider specific recommendations, such as subsidizing healthy foods and creating safe spaces for physical activity, to facilitate healthier behaviors. Another approach is to establish a national registry for T2D and CVD patients that can help track health outcomes and inform future interventions. Also, collaborative research initiatives with academic institutions should be prioritized to evaluate the effectiveness of existing programs and identify barriers to implementation. Finally, culturally sensitive approaches are vital to ensure that interventions align with Qatar's diverse population.

Our study's methodology possesses a primary strength in its use of a dynamic population model coupled with the PALYs metric. Unlike traditional static life table models, this dynamic approach considers factors such as demographic shifts and disease incidence, allowing for a more realistic projection of outcomes. This is a significant advantage as it provides a more accurate representation of the healthcare and societal burden of CVD among people with T2D in Qatar. Furthermore, the model's stratification of primary and secondary prevention populations highlights the substantial productivity and costs involved in chronic disease management and treatment. Also, our methodology considers different employment status categories, including those who are employed, unemployed, and not in the labor force, improving the evaluation of outcomes and allowing for a more holistic analysis. Moreover, further contributing to strength and validity, the model's use of Qatari age- and sex-specific data, as well as the robustness of the data sources utilized.

However, this study acknowledges several limitations. While the analysis provides valuable national-level insights and forecasts on the PALYs and economic burden of CVD risk in T2D in Qatar, the accuracy and performance of the 2013 PCE-ASCVD algorithm used have not been externally validated in the Qatari population. There have been no studies assessing the validity and applicability of long-term CVD risk prediction models in an Arab population, except for a study in the United Arab Emirates [31], which found that the 2013 PCE-ASCVD model and Framingham models overestimated CVD risk. It is worth mentioning that calculating a 10-year CVD risk and validating the algorithm in Qatar is currently challenging as it has not been 10 years since the adoption of electronic medical records in practices. The reliance on an unvalidated algorithm may lead to inflated estimates of the CVD burden, which could misguide public health strategies and resource allocation. In addition, the applicability of the PCE-ASCVD model may not fully capture unique risk factors prevalent in the Arab population, potentially significantly impacting CVD risk assessments. In any case, it must be noted here that the 2013 PCE-ASCVD model is currently the only one used in clinical practice for predicting CVD risk in Qatar. Also, a limitation is the reliance on estimates from the global REACH registry for recurrent CVD events. The absence of locally specific data regarding recurrent MI and stroke in individuals with existing MI or stroke or both, may affect the accuracy of our model. Also, the REACH registry is limited to a follow-up period of only four years, although it includes participants from the MENA region. Another limitation is that the model does not capture other CVD comorbidities. This is primarily due to using the PCE algorithm, which only includes MI and stroke as specific endpoints for CVD risk assessment. As a result, our model may underestimate the true actual burden. Another limitation that may also underestimate findings is that our model only considered Qataris aged 40 to 65 years with T2D. However, T2D and CVD are relatively uncommon among people under the age of 40. Moreover, the model's assumption of sustained improvements in risk factor control throughout the time horizon may also be unrealistic. The model also did not account for potential adverse outcomes, such as hypotension from intensive reduction in hypertension, or the resources and costs required to achieve the interventional improvements. Finally, the projections of GDP were based solely on productivity-related factors and did not include the direct medical costs of managing CVD.

In conclusion, while the findings of the present study highlight the potential significant impact of CVD on PALYs in the Qatari population with T2D in the coming decade, they articulate that there are real opportunities to mitigate this burden through improved control of CVD risk factors.

## Source of funding

Publication fees is provided through Qatar University**.**

## CRediT authorship contribution statement

**Dina Abushanab:** Writing – review & editing, Writing – original draft, Visualization, Validation, Supervision, Software, Resources, Project administration, Methodology, Investigation, Formal analysis, Data curation. **Daoud Al-Badriyeh:** Writing – review & editing, Validation, Supervision, Project administration, Methodology, Investigation, Funding acquisition, Conceptualization. **Danny Liew:** Writing – review & editing, Supervision, Investigation. **Zanfina Ademi:** Writing – review & editing, Validation, Supervision, Project administration, Methodology, Investigation, Conceptualization.

## Declaration of competing interest

All authors declare that they have no known competing financial interests or personal relationships that could have appeared to influence the work reported in this paper.

## Appendix Supplementary materials

Image, application 1
