# Supplementary material for: Unraveling the future productivity burden of cardiovascular disease in Qatar: Investigating the modifiable risk factors control in type 2 diabetes
Source: Am J Prev Cardiol. 2025 Mar 11;22:100961. doi: 10.1016/j.ajpc.2025.100961 (PMC11999317; doi:10.1016/j.ajpc.2025.100961)
Supplement: Supplementary file 1 [file mmc1.docx]

**Full title:** Unraveling the Future Productivity Burden of Cardiovascular Disease in Qatar: Investigating the Modifiable Risk Factors Control in Type 2 Diabetes.

**Authors:**

Dina Abushanab^1,2*^, Daoud Al-Badriyeh^3*^, Danny Liew^4^, Zanfina Ademi^1,5^

**Affiliations:**

^1^Health Economics and Policy Evaluation Research (HEPER), Centre for Medicine Use and Safety, Faculty of Pharmacy and Pharmaceutical Sciences, Monash University, Melbourne, Australia.

^2^Department of Pharmacy, Hamad Medical Corporation, Doha, Qatar.

^3^College of Pharmacy, QU Health, Qatar University, Doha, Qatar.

^4^The Adelaide Medical School, The University of Adelaide, Adelaide, Australia.

^5^School of Public Health and Preventive Medicine, Monash University, Melbourne, Australia.

**Corresponding authors**

Dina Abushanab, MSc, Health Economics and Policy Evaluation Research (HEPER) Group Centre for Medicine Use and Safety, Faculty of Pharmacy and Pharmaceutical Sciences, Monash University, Melbourne, Australia. Department of Pharmacy, Hamad Medical Corporation, Doha, Qatar. Email: [dina.abushanab@monash.edu](mailto:dina.abushanab@monash.edu).

Daoud Al-Badriyeh, PhD, Professor, College of Pharmacy, QU Health, Qatar University, Doha, Qatar. Email: [daoud.a@qu.edu.qa](mailto:daoud.a@qu.edu.qa).

**Supplementary Appendices**

Supplementary Table 1: Prevalence and Incidence of Type 2 Diabetes by Age and Sex in the Qatari Population, and Number of People Affected by Type 2 Diabetes.
Supplementary Table 2: Prevalence of Coronary Heart Disease and Stroke by Age and Sex in the Qatari Population.
Supplementary Table 3: Projections of Migration among People with Type 2 Diabetes by Age and Sex.
Supplementary Table 4: Projections of Mortality among People with Type 2 Diabetes by Age and Sex.
Supplementary Table 5: Description of Model Inputs and Data Sources.
Supplementary Table 6: Description of Data Sources Used in the Intervention Scenarios.

Supplementary Table 7. Model Input Variables and Distributions.
Supplementary Table 8: Impact on Years of Life Lived in a Qatari Working-Age Cohort with Type 2 Diabetes and First Cardiovascular Disease Events.
Supplementary Table 9: Impact on Productivity-Adjusted Life Years (PALYs) in a Qatari Working-Age Cohort with Type 2 Diabetes and First Cardiovascular Disease Events.
Supplementary Table 10: Impact on Cost of Productivity-Adjusted Life Years (PALYs) in a Qatari Working-Age Cohort with Type 2 Diabetes and First Cardiovascular Disease Events.
Supplementary Table 11: Impact on Years of Life Lived in a Qatari Working-Age Cohort with Type 2 Diabetes and Recurrent Cardiovascular Disease Events.
Supplementary Table 12: Impact on Productivity-Adjusted Life Years (PALYs) in a Qatari Working-Age Cohort with Type 2 Diabetes and Recurrent Cardiovascular Disease Events.
Supplementary Table 13: Impact on Cost of Productivity-Adjusted Life Years (PALYs) in a Qatari Working-Age Cohort with Type 2 Diabetes and Recurrent Cardiovascular Disease Events.
Supplementary Table 14: Impact on Years of Life Lived in a Qatari Working-Age Cohort with Type 2 Diabetes and First and Recurrent Cardiovascular Disease Events.
Supplementary Table 15: Impact on Productivity-Adjusted Life Years (PALYs) in a Qatari Working-Age Cohort with Type 2 Diabetes and First and Recurrent Cardiovascular Disease Events.
Supplementary Table 16: Impact on Cost of Productivity-Adjusted Life Years (PALYs) in a Qatari Working-Age Cohort with Type 2 Diabetes and First and Recurrent Cardiovascular Disease Events.
Supplementary Table 17: Assessment of the Validation Status of Health-Economic Decision Models.

**Supplementary Table 1: Prevalence and Incidence of Type 2 Diabetes by Age and Sex in the Qatari Population, and Number of People Affected by Type 2 Diabetes.**

|  | **Men** | | | | **Women** | | | |
| --- | --- | --- | --- | --- | --- | --- | --- | --- |
| **Age** | **Total general population** | **Prevalence rate of type 2 diabetes** | **Total population with type 2 diabetes** | **Incidence rate of type 2 diabetes** | **Total general population** | **Prevalence rate of type 2 diabetes** | **Total population with type 2 diabetes** | **Incidence rate of type 2 diabetes** |
| 40 | 72,075 | 35.69% | 25,725 | 8.7% | 18,001 | 32.90% | 5,922 | 32.4% |
| 41 | 68,109 | 35.92% | 24,463 | 8.7% | 17,506 | 33.44% | 5,854 | 32.4% |
| 42 | 63,879 | 36.13% | 23,080 | 8.7% | 16,906 | 33.96% | 5,741 | 32.4% |
| 43 | 59,516 | 36.33% | 21,621 | 8.7% | 16,214 | 34.46% | 5,587 | 32.4% |
| 44 | 55,150 | 36.51% | 20,137 | 8.7% | 15,443 | 34.94% | 5,396 | 32.4% |
| 45 | 41,841 | 36.68% | 15,348 | 29.6% | 13,645 | 35.40% | 4,830 | 43.5% |
| 46 | 38,570 | 36.84% | 14,208 | 29.6% | 12,818 | 35.84% | 4,594 | 43.5% |
| 47 | 35,618 | 36.98% | 13,172 | 29.6% | 11,956 | 36.26% | 4,335 | 43.5% |
| 48 | 33,092 | 37.11% | 12,280 | 29.6% | 11,072 | 36.66% | 4,059 | 43.5% |
| 49 | 31,099 | 37.22% | 11,576 | 29.6% | 10,179 | 37.04% | 3,770 | 43.5% |
| 50 | 35,068 | 37.32% | 13,088 | 29.6% | 9,528 | 37.40% | 3,563 | 43.5% |
| 51 | 34,355 | 37.41% | 12,851 | 29.6% | 8,633 | 37.74% | 3,258 | 43.5% |
| 52 | 34,648 | 37.48% | 12,986 | 29.6% | 7,769 | 38.06% | 2,957 | 43.5% |
| 53 | 36,076 | 37.54% | 13,542 | 29.6% | 6,947 | 38.36% | 2,665 | 43.5% |
| 54 | 38,763 | 37.58% | 14,568 | 29.6% | 6,183 | 38.64% | 2,389 | 43.5% |
| 55 | 22,807 | 37.61% | 8,578 | 39% | 5,890 | 38.90% | 2,291 | 30.4% |
| 56 | 21,368 | 37.63% | 8,040 | 39% | 5,233 | 39.14% | 2,048 | 30.4% |
| 57 | 19,962 | 37.63% | 7,512 | 39% | 4,679 | 39.36% | 1,842 | 30.4% |
| 58 | 18,588 | 37.62% | 6,992 | 39% | 4,243 | 39.56% | 1,678 | 30.4% |
| 59 | 17,247 | 37.59% | 6,483 | 39% | 3,938 | 39.74% | 1,565 | 30.4% |
| 60 | 15,399 | 37.55% | 5,783 | 39% | 3,050 | 39.90% | 1,217 | 30.4% |
| 61 | 14,167 | 37.50% | 5,312 | 39% | 3,050 | 40.04% | 1,221 | 30.4% |
| 62 | 12,966 | 37.43% | 4,853 | 39% | 3,190 | 40.16% | 1,281 | 30.4% |
| 63 | 11,797 | 37.35% | 4,406 | 39% | 3,482 | 40.26% | 1,402 | 30.4% |
| 64 | 10,660 | 37.25% | 3,971 | 39% | 3,937 | 40.34% | 1,588 | 30.4% |
| 65 | 8,623 | 37.14% | 3,203 | 24% | 2,409 | 40.40% | 973 | 24.3% |

**Supplementary Table 2: Prevalence of Coronary Heart Disease and Stroke by Age and Sex in the Qatari Population.**

|  | **Men** | | | | | **Women** | | | | | |
| --- | --- | --- | --- | --- | --- | --- | --- | --- | --- | --- | --- |
| **Age** | **CHD, Prevalence %** | **Stroke, Prevalence %** | **Total population with type 2 diabetes and without CVD** | **Total population with type 2 diabetes and with CHD** | **Total population with type 2 diabetes and with stroke** | | **CHD, Prevalence %** | **Stroke, Prevalence %** | **Total population with type 2 diabetes and without CVD** | **Total population with type 2 diabetes and with CHD** | **Total population with type 2 diabetes and with stroke** |
| 40 | 0.35% | 0.35% | 25,545 | 90 | 90 | | 0.06% | 0.03% | 5,917 | 3 | 1 |
| 41 | 0.43% | 0.38% | 24,264 | 106 | 93 | | 0.07% | 0.03% | 5,848 | 4 | 2 |
| 42 | 0.54% | 0.41% | 22,861 | 124 | 95 | | 0.10% | 0.03% | 5,734 | 6 | 2 |
| 43 | 0.65% | 0.44% | 21,383 | 141 | 96 | | 0.12% | 0.03% | 5,579 | 7 | 2 |
| 44 | 0.79% | 0.48% | 19,881 | 159 | 97 | | 0.15% | 0.04% | 5,386 | 8 | 2 |
| 45 | 0.94% | 0.52% | 15,125 | 144 | 79 | | 0.19% | 0.04% | 4,819 | 9 | 2 |
| 46 | 1.11% | 0.56% | 13,972 | 158 | 79 | | 0.23% | 0.04% | 4,581 | 11 | 2 |
| 47 | 1.29% | 0.60% | 12,922 | 170 | 79 | | 0.28% | 0.05% | 4,321 | 12 | 2 |
| 48 | 1.50% | 0.64% | 12,017 | 184 | 79 | | 0.32% | 0.05% | 4,044 | 13 | 2 |
| 49 | 1.71% | 0.69% | 11,298 | 198 | 79 | | 0.38% | 0.06% | 3,754 | 14 | 2 |
| 50 | 1.95% | 0.73% | 12,737 | 255 | 96 | | 0.44% | 0.06% | 3,546 | 16 | 2 |
| 51 | 2.20% | 0.78% | 12,468 | 283 | 101 | | 0.50% | 0.07% | 3,240 | 16 | 2 |
| 52 | 2.47% | 0.84% | 12,557 | 320 | 109 | | 0.57% | 0.07% | 2,938 | 17 | 2 |
| 53 | 2.75% | 0.89% | 13,049 | 373 | 121 | | 0.64% | 0.08% | 2,646 | 17 | 2 |
| 54 | 3.05% | 0.95% | 13,985 | 445 | 138 | | 0.72% | 0.09% | 2,370 | 17 | 2 |
| 55 | 3.37% | 1.01% | 8,203 | 289 | 86 | | 0.80% | 0.10% | 2,271 | 18 | 2 |
| 56 | 3.71% | 1.07% | 7,656 | 298 | 86 | | 0.89% | 0.10% | 2,028 | 18 | 2 |
| 57 | 4.06% | 1.14% | 7,122 | 305 | 85 | | 0.98% | 0.11% | 1,822 | 18 | 2 |
| 58 | 4.42% | 1.20% | 6,599 | 309 | 84 | | 1.07% | 0.13% | 1,658 | 18 | 2 |
| 59 | 4.81% | 1.28% | 6,089 | 312 | 83 | | 1.17% | 0.14% | 1,544 | 18 | 2 |
| 60 | 5.21% | 1.35% | 5,403 | 301 | 78 | | 1.28% | 0.15% | 1,200 | 16 | 2 |
| 61 | 5.63% | 1.43% | 4,938 | 299 | 76 | | 1.39% | 0.16% | 1,202 | 17 | 2 |
| 62 | 6.06% | 1.51% | 4,486 | 294 | 73 | | 1.50% | 0.18% | 1,260 | 19 | 2 |
| 63 | 6.51% | 1.59% | 4,049 | 287 | 70 | | 1.62% | 0.20% | 1,376 | 23 | 3 |
| 64 | 6.98% | 1.67% | 3,628 | 277 | 67 | | 1.74% | 0.21% | 1,557 | 28 | 3 |
| 65 | 7.46% | 1.76% | 2,907 | 239 | 56 | | 1.87% | 0.23% | 953 | 18 | 2 |

*CHD: coronary heart disease.

**Supplementary Table 3: Projections of Migration among People with Type 2 Diabetes by Age and Sex.**

| **Men** | | | | | | | | | | |
| --- | --- | --- | --- | --- | --- | --- | --- | --- | --- | --- |
| **Age** | **2024** | **2025** | **2026** | **2027** | **2028** | **2029** | **2030** | **2031** | **2032** | **2033** |
| 40 | 455 | 431 | 428 | 408 | 360 | 343 | 326 | 309 | 292 | 292 |
| 41 | 458 | 434 | 430 | 410 | 362 | 345 | 328 | 311 | 294 | 294 |
| 42 | 461 | 437 | 433 | 413 | 364 | 347 | 330 | 313 | 296 | 296 |
| 43 | 463 | 439 | 435 | 415 | 366 | 349 | 332 | 315 | 298 | 298 |
| 44 | 466 | 441 | 438 | 417 | 368 | 351 | 334 | 316 | 299 | 299 |
| 45 | 301 | 285 | 283 | 270 | 238 | 227 | 216 | 205 | 193 | 193 |
| 46 | 302 | 287 | 284 | 271 | 239 | 228 | 217 | 205 | 194 | 194 |
| 47 | 304 | 288 | 285 | 272 | 240 | 229 | 218 | 206 | 195 | 195 |
| 48 | 305 | 289 | 286 | 273 | 241 | 230 | 218 | 207 | 196 | 196 |
| 49 | 305 | 290 | 287 | 274 | 242 | 230 | 219 | 208 | 196 | 196 |
| 50 | 164 | 156 | 155 | 147 | 130 | 124 | 118 | 112 | 106 | 106 |
| 51 | 165 | 156 | 155 | 148 | 130 | 124 | 118 | 112 | 106 | 106 |
| 52 | 165 | 156 | 155 | 148 | 131 | 124 | 118 | 112 | 106 | 106 |
| 53 | 165 | 157 | 155 | 148 | 131 | 125 | 119 | 112 | 106 | 106 |
| 54 | 166 | 157 | 156 | 148 | 131 | 125 | 119 | 113 | 106 | 106 |
| 55 | 86 | 82 | 81 | 76 | 68 | 65 | 62 | 59 | 55 | 55 |
| 56 | 86 | 82 | 81 | 76 | 68 | 65 | 62 | 59 | 55 | 55 |
| 57 | 86 | 82 | 81 | 76 | 68 | 65 | 62 | 59 | 55 | 55 |
| 58 | 86 | 82 | 81 | 76 | 68 | 65 | 62 | 59 | 55 | 55 |
| 59 | 86 | 82 | 81 | 76 | 68 | 65 | 62 | 59 | 55 | 55 |
| 60 | 21 | 20 | 20 | 19 | 16 | 16 | 15 | 14 | 13 | 13 |
| 61 | 21 | 20 | 20 | 19 | 16 | 16 | 15 | 14 | 13 | 13 |
| 62 | 21 | 20 | 20 | 19 | 16 | 16 | 15 | 14 | 13 | 13 |
| 63 | 21 | 20 | 19 | 19 | 16 | 16 | 15 | 14 | 13 | 13 |
| 64 | 21 | 20 | 19 | 19 | 16 | 16 | 15 | 14 | 13 | 13 |
| 65 | 15 | 15 | 14 | 14 | 12 | 12 | 11 | 10 | 10 | 10 |
| **Women** | | | | | | | | | | |
| **Age** | **2024** | **2025** | **2026** | **2027** | **2028** | **2029** | **2030** | **2031** | **2032** | **2033** |
| 40 | 87 | 87 | 87 | 87 | 87 | 87 | 87 | 87 | 87 | 87 |
| 41 | 89 | 89 | 89 | 89 | 89 | 89 | 89 | 89 | 89 | 89 |
| 42 | 90 | 90 | 90 | 90 | 90 | 90 | 90 | 90 | 90 | 90 |
| 43 | 91 | 91 | 91 | 91 | 91 | 91 | 91 | 91 | 91 | 91 |
| 44 | 93 | 93 | 93 | 93 | 93 | 93 | 93 | 93 | 93 | 93 |
| 45 | 62 | 62 | 62 | 62 | 62 | 62 | 62 | 62 | 62 | 62 |
| 46 | 63 | 63 | 63 | 63 | 63 | 63 | 63 | 63 | 63 | 63 |
| 47 | 64 | 64 | 64 | 64 | 64 | 64 | 64 | 64 | 64 | 64 |
| 48 | 65 | 65 | 65 | 65 | 65 | 65 | 65 | 65 | 65 | 65 |
| 49 | 65 | 65 | 65 | 65 | 65 | 65 | 65 | 65 | 65 | 65 |
| 50 | 39 | 39 | 39 | 39 | 39 | 39 | 39 | 39 | 39 | 39 |
| 51 | 39 | 39 | 39 | 39 | 39 | 39 | 39 | 39 | 39 | 39 |
| 52 | 39 | 39 | 39 | 39 | 39 | 39 | 39 | 39 | 39 | 39 |
| 53 | 40 | 40 | 40 | 40 | 40 | 40 | 40 | 40 | 40 | 40 |
| 54 | 40 | 40 | 40 | 40 | 40 | 40 | 40 | 40 | 40 | 40 |
| 55 | 103 | 103 | 103 | 103 | 103 | 103 | 103 | 103 | 103 | 103 |
| 56 | 104 | 104 | 104 | 104 | 104 | 104 | 104 | 104 | 104 | 104 |
| 57 | 105 | 105 | 105 | 105 | 105 | 105 | 105 | 105 | 105 | 105 |
| 58 | 105 | 105 | 105 | 105 | 105 | 105 | 105 | 105 | 105 | 105 |
| 59 | 106 | 106 | 106 | 106 | 106 | 106 | 106 | 106 | 106 | 106 |
| 60 | 8 | 8 | 8 | 8 | 8 | 8 | 8 | 8 | 8 | 8 |
| 61 | 8 | 8 | 8 | 8 | 8 | 8 | 8 | 8 | 8 | 8 |
| 62 | 8 | 8 | 8 | 8 | 8 | 8 | 8 | 8 | 8 | 8 |
| 63 | 8 | 8 | 8 | 8 | 8 | 8 | 8 | 8 | 8 | 8 |
| 64 | 9 | 9 | 9 | 9 | 9 | 9 | 9 | 9 | 9 | 9 |
| 65 | 8 | 8 | 8 | 8 | 8 | 8 | 8 | 8 | 8 | 8 |

**Supplementary Table 4: Projections of Mortality among People with Type 2 Diabetes by Age and Sex.**

| **Men** | | | | | | | | | | |
| --- | --- | --- | --- | --- | --- | --- | --- | --- | --- | --- |
| **Age** | **2024** | **2025** | **2026** | **2027** | **2028** | **2029** | **2030** | **2031** | **2032** | **2033** |
| 40 | 0.00137 | 0.00136 | 0.00136 | 0.00136 | 0.00135 | 0.00135 | 0.00135 | 0.00135 | 0.00137 | 0.00044 |
| 41 | 0.00149 | 0.00149 | 0.00148 | 0.00148 | 0.00147 | 0.00147 | 0.00147 | 0.00147 | 0.00149 | 0.00047 |
| 42 | 0.00160 | 0.00160 | 0.00159 | 0.00159 | 0.00159 | 0.00158 | 0.00158 | 0.00158 | 0.00161 | 0.00049 |
| 43 | 0.00172 | 0.00172 | 0.00171 | 0.00171 | 0.00171 | 0.00171 | 0.00170 | 0.00170 | 0.00173 | 0.00052 |
| 44 | 0.00183 | 0.00182 | 0.00182 | 0.00181 | 0.00181 | 0.00181 | 0.00181 | 0.00181 | 0.00183 | 0.00055 |
| 45 | 0.00195 | 0.00194 | 0.00194 | 0.00194 | 0.00194 | 0.00193 | 0.00193 | 0.00193 | 0.00195 | 0.00058 |
| 46 | 0.00211 | 0.00210 | 0.00210 | 0.00210 | 0.00210 | 0.00209 | 0.00209 | 0.00209 | 0.00211 | 0.00061 |
| 47 | 0.00228 | 0.00228 | 0.00228 | 0.00227 | 0.00227 | 0.00227 | 0.00227 | 0.00226 | 0.00229 | 0.00065 |
| 48 | 0.00249 | 0.00248 | 0.00248 | 0.00248 | 0.00247 | 0.00247 | 0.00247 | 0.00247 | 0.00249 | 0.00068 |
| 49 | 0.00270 | 0.00269 | 0.00269 | 0.00269 | 0.00268 | 0.00268 | 0.00268 | 0.00267 | 0.00270 | 0.00072 |
| 50 | 0.00291 | 0.00290 | 0.00289 | 0.00289 | 0.00288 | 0.00288 | 0.00288 | 0.00287 | 0.00291 | 0.00076 |
| 51 | 0.00314 | 0.00313 | 0.00312 | 0.00312 | 0.00311 | 0.00311 | 0.00311 | 0.00310 | 0.00315 | 0.00080 |
| 52 | 0.00339 | 0.00338 | 0.00337 | 0.00336 | 0.00336 | 0.00335 | 0.00334 | 0.00334 | 0.00339 | 0.00085 |
| 53 | 0.00367 | 0.00366 | 0.00365 | 0.00364 | 0.00364 | 0.00363 | 0.00362 | 0.00362 | 0.00368 | 0.00090 |
| 54 | 0.00399 | 0.00398 | 0.00397 | 0.00396 | 0.00396 | 0.00395 | 0.00394 | 0.00393 | 0.00401 | 0.00095 |
| 55 | 0.00434 | 0.00433 | 0.00431 | 0.00431 | 0.00430 | 0.00429 | 0.00428 | 0.00427 | 0.00435 | 0.00100 |
| 56 | 0.00470 | 0.00468 | 0.00467 | 0.00466 | 0.00465 | 0.00464 | 0.00463 | 0.00462 | 0.00471 | 0.00105 |
| 57 | 0.00508 | 0.00507 | 0.00505 | 0.00504 | 0.00503 | 0.00502 | 0.00501 | 0.00499 | 0.00510 | 0.00111 |
| 58 | 0.00549 | 0.00547 | 0.00545 | 0.00543 | 0.00542 | 0.00541 | 0.00539 | 0.00538 | 0.00551 | 0.00117 |
| 59 | 0.00592 | 0.00589 | 0.00586 | 0.00585 | 0.00583 | 0.00581 | 0.00580 | 0.00578 | 0.00594 | 0.00124 |
| 60 | 0.00637 | 0.00634 | 0.00630 | 0.00628 | 0.00626 | 0.00624 | 0.00622 | 0.00620 | 0.00641 | 0.00131 |
| 61 | 0.00684 | 0.00680 | 0.00675 | 0.00673 | 0.00670 | 0.00668 | 0.00665 | 0.00663 | 0.00688 | 0.00138 |
| 62 | 0.00734 | 0.00729 | 0.00723 | 0.00720 | 0.00717 | 0.00714 | 0.00711 | 0.00708 | 0.00739 | 0.00146 |
| 63 | 0.00789 | 0.00783 | 0.00777 | 0.00773 | 0.00769 | 0.00766 | 0.00762 | 0.00759 | 0.00795 | 0.00154 |
| 64 | 0.00851 | 0.00843 | 0.00836 | 0.00832 | 0.00828 | 0.00824 | 0.00820 | 0.00815 | 0.00858 | 0.00163 |
| 65 | 0.00918 | 0.00910 | 0.00902 | 0.00897 | 0.00892 | 0.00887 | 0.00883 | 0.00878 | 0.00927 | 0.00172 |
| **Women** | | | | | | | | | | |
| **Age** | **2024** | **2025** | **2026** | **2027** | **2028** | **2029** | **2030** | **2031** | **2032** | **2033** |
| 40 | 0.00077 | 0.00077 | 0.00077 | 0.00077 | 0.00077 | 0.00077 | 0.00077 | 0.00076 | 0.00076 | 0.00025 |
| 41 | 0.00084 | 0.00084 | 0.00084 | 0.00084 | 0.00084 | 0.00083 | 0.00083 | 0.00083 | 0.00083 | 0.00026 |
| 42 | 0.00092 | 0.00092 | 0.00092 | 0.00091 | 0.00091 | 0.00091 | 0.00091 | 0.00091 | 0.00091 | 0.00027 |
| 43 | 0.00101 | 0.00101 | 0.00100 | 0.00100 | 0.00100 | 0.00100 | 0.00100 | 0.00100 | 0.00100 | 0.00029 |
| 44 | 0.00110 | 0.00109 | 0.00109 | 0.00109 | 0.00109 | 0.00109 | 0.00109 | 0.00109 | 0.00109 | 0.00031 |
| 45 | 0.00119 | 0.00119 | 0.00119 | 0.00119 | 0.00119 | 0.00119 | 0.00119 | 0.00119 | 0.00118 | 0.00032 |
| 46 | 0.00129 | 0.00129 | 0.00129 | 0.00129 | 0.00129 | 0.00128 | 0.00128 | 0.00128 | 0.00128 | 0.00034 |
| 47 | 0.00140 | 0.00140 | 0.00139 | 0.00139 | 0.00139 | 0.00139 | 0.00139 | 0.00139 | 0.00139 | 0.00036 |
| 48 | 0.00150 | 0.00150 | 0.00150 | 0.00150 | 0.00150 | 0.00150 | 0.00149 | 0.00149 | 0.00149 | 0.00038 |
| 49 | 0.00163 | 0.00163 | 0.00162 | 0.00162 | 0.00162 | 0.00162 | 0.00162 | 0.00162 | 0.00162 | 0.00041 |
| 50 | 0.00176 | 0.00176 | 0.00176 | 0.00176 | 0.00175 | 0.00175 | 0.00175 | 0.00175 | 0.00175 | 0.00043 |
| 51 | 0.00192 | 0.00191 | 0.00191 | 0.00191 | 0.00191 | 0.00190 | 0.00190 | 0.00190 | 0.00190 | 0.00045 |
| 52 | 0.00209 | 0.00209 | 0.00208 | 0.00208 | 0.00208 | 0.00207 | 0.00207 | 0.00207 | 0.00207 | 0.00048 |
| 53 | 0.00227 | 0.00227 | 0.00226 | 0.00226 | 0.00225 | 0.00225 | 0.00225 | 0.00224 | 0.00224 | 0.00051 |
| 54 | 0.00245 | 0.00244 | 0.00244 | 0.00243 | 0.00243 | 0.00243 | 0.00242 | 0.00242 | 0.00242 | 0.00054 |
| 55 | 0.00264 | 0.00263 | 0.00262 | 0.00262 | 0.00261 | 0.00261 | 0.00261 | 0.00260 | 0.00260 | 0.00057 |
| 56 | 0.00283 | 0.00282 | 0.00282 | 0.00281 | 0.00281 | 0.00280 | 0.00280 | 0.00279 | 0.00279 | 0.00060 |
| 57 | 0.00305 | 0.00304 | 0.00302 | 0.00302 | 0.00301 | 0.00301 | 0.00300 | 0.00300 | 0.00299 | 0.00063 |
| 58 | 0.00327 | 0.00326 | 0.00325 | 0.00324 | 0.00324 | 0.00323 | 0.00322 | 0.00321 | 0.00321 | 0.00067 |
| 59 | 0.00353 | 0.00351 | 0.00350 | 0.00349 | 0.00348 | 0.00347 | 0.00347 | 0.00346 | 0.00345 | 0.00071 |
| 60 | 0.00380 | 0.00378 | 0.00376 | 0.00375 | 0.00374 | 0.00373 | 0.00372 | 0.00371 | 0.00371 | 0.00075 |
| 61 | 0.00407 | 0.00405 | 0.00403 | 0.00402 | 0.00401 | 0.00400 | 0.00399 | 0.00397 | 0.00396 | 0.00079 |
| 62 | 0.00438 | 0.00435 | 0.00433 | 0.00431 | 0.00430 | 0.00428 | 0.00427 | 0.00426 | 0.00425 | 0.00084 |
| 63 | 0.00472 | 0.00469 | 0.00466 | 0.00465 | 0.00463 | 0.00461 | 0.00460 | 0.00458 | 0.00457 | 0.00089 |
| 64 | 0.00511 | 0.00507 | 0.00504 | 0.00502 | 0.00500 | 0.00498 | 0.00497 | 0.00495 | 0.00493 | 0.00094 |
| 65 | 0.00556 | 0.00552 | 0.00548 | 0.00545 | 0.00543 | 0.00541 | 0.00539 | 0.00537 | 0.00535 | 0.00099 |

**Supplementary Table 5. Description of Model Inputs and Data Sources.**

| **No.** | **Input** | **Description** | **Data source** |
| --- | --- | --- | --- |
| **1** | The population | The model population utilized in this study was derived from the Institute for Health Metrics and Evaluation, using data from the year 2023. | The Institute for Health Metrics and Evaluation is an international organization dedicated to population health research, specializing in global health statistics and impact evaluation (1). |
| **2** | Prevalence of type 2 diabetes | The latest available data for the age- and sex-specific prevalence of type 2 diabetes in Qatar is based on the study conducted by Awad et al. This study utilized a population-level mathematical model to estimate the prevalence of type 2 diabetes in Qatar for the year 2019. | Awad et al. 2019 (2). |
| **3** | Incidence of type 2 diabetes | The incidence of type 2 diabetes in Qatar, specifically in relation to age and sex, was examined in a study conducted by Bener et al. This study utilized a prediction model to estimate the incidence of type 2 diabetes in Qatar. It was an observational cohort study that relied on data obtained from the registry at Hamad General Hospital and Primary Health Care (PHC) centers in the State of Qatar. The study included patients above the age of 25 who were diagnosed with type 2 diabetes between January 2004 and July 2014. | Bener et al. 2016 (3). |
| **4** | Prevalence of CVD (non-fatal MI and stroke) | The estimates of age- and sex-specific prevalence of established cardiovascular disease (CVD), specifically non-fatal myocardial infarction (MI) and stroke, in the population of Qatar were derived from data obtained from the study conducted by Syed et al. This study, which was a cross-sectional study, focused on individuals aged 18 years and older who sought healthcare services from publicly funded primary care facilities in Qatar during the year 2017. The aim of the study was to determine the prevalence of non-communicable diseases, including CVD. | Syed et al. 2019 (4). |
| **5** | Age- and sex-specific CVD risk estimates in people with type 2 diabetes | The assessment of incident cardiovascular disease (CVD) risk among individuals with type 2 diabetes but without prior CVD was conducted using the 2013 PCE-ASCVD tool (5). This tool was specifically designed to estimate the 10-year absolute rate of incident CVD, taking into account factors such as sex, age, race, smoking status, systolic blood pressure, diabetes status, hypertension treatment status, and total and high-density lipoprotein cholesterol levels.  To calculate the risk estimates, individual parameters were extracted from the latest available data spanning from 2016 to 2021. This data was obtained from the principal provider of primary care in Qatar, which operates 31 clinics across the country. The dataset comprised a total of 48,009 Qataris with type 2 diabetes, aged between 40 and 79 years. | Data from the PHCC 2016-2021 (6). |
| **6** | Proportions of CVD events | To determine the proportions of fatal and non-fatal myocardial infarction (MI) and stroke events among individuals with type 2 diabetes in Qatar, we relied on local studies as the 2013 PCE-ASCVD equation only considers combined composite cardiovascular disease (CVD) outcomes.  Based on the estimates obtained from these studies, the proportions were found to be as follows:   - For MI events: The proportion of fatal events was calculated to be 0.17, while the proportion of non-fatal events was estimated to be 0.83. - For stroke events: The proportion of fatal stroke events was determined to be 0.01, while the proportion of non-fatal stroke events was estimated to be 0.99. | Syed et al. (2019) (4), El-Menyar et al. (2009) (7), and Khan et al. (2008) (8). |
| **7** | Migration data | The data regarding overall net migration in Qatar was sourced from the World Bank Database. Specifically, the projected number of immigrants and emigrants was categorized by sex and single year of age. This data was meticulously adjusted to account for individuals with type 2 diabetes (T2D). By incorporating this adjusted data, a more accurate picture of the migration patterns in relation to T2D in Qatar can be obtained. | World Bank Database (9). |
| **8** | Mortality data | The mortality data, categorized by sex and single year of age, in Qatar was obtained from a reputable global database known as CEIC. This comprehensive database provides valuable insights into mortality rates. To ensure accuracy, the data was carefully adjusted to account for individuals with type 2 diabetes (T2D). By incorporating this adjustment, we can gain a more precise understanding of mortality patterns in relation to T2D in Qatar. | CEIC, a trusted source of economic and financial data, provides economists and investment professionals with comprehensive and relevant information to effectively monitor and gain genuine insights into market dynamics. By curating the best available data, CEIC enables users to stay informed about the latest trends and developments in their respective fields. This empowers economists and investment professionals to make informed decisions and navigate the complexities of their markets with confidence (10). |
| **9** | Proportions of recurrent fatal and non-fatal CVD in people with type 2 diabetes and established CVD | The input probabilities of recurrent myocardial infarction (MI), stroke, and cardiovascular disease (CVD) death among individuals with both type 2 diabetes (T2D) and CVD were obtained from the esteemed Reduction of Atherothrombosis for Continued Health (REACH) registry. The REACH registry is a global prospective observational longitudinal study that focuses on individuals with, or at high risk of, atherothrombosis. Within this registry, a substantial subset of 30,043 individuals had a diagnosis of diabetes. This valuable data from the REACH registry allows for a more comprehensive understanding of the probabilities associated with recurrent MI, stroke, and CVD death in individuals with T2D and CVD. | REACH registry (11). |
| **10** | The number of days absent from work due to MI and stroke | Data from a large national sample, representing approximately 40% of the Danish population, was utilized to identify a cohort of 34,882 individuals aged 18 to 70 years with hospital-diagnosed diabetes. This cohort was carefully selected based on six years of comprehensive hospital utilization data. By drawing from this extensive registry, we were able to capture a significant portion of the Danish population and gain valuable insights into the healthcare utilization patterns of individuals with diabetes. | Sørensen J and Jon Ploug et al. 2013 (12) |
| **11** | The number of days absent from work in people without the disease | To investigate the impact of type 2 diabetes on lost productivity, including absenteeism, presenteeism, and early retirement, a systematic review was conducted. This review aimed to compare the productivity outcomes of individuals with type 2 diabetes to those without the disease.  The absenteeism data for individuals with T2D due to MI and stroke were sourced from a study by Sørensen and Jon Ploug (12). In the initial year, individuals with T2D had 92 days of work absence due to MI, decreasing to 56 days in subsequent years. For stroke, the individuals experienced 125 absent days in the first year and 71 days in the following years. In contrast, those without MI or stroke had 38 absent days in both the first and subsequent years. | Breton et al. 2013 (13) |
| **12** | The number of days of unproductive time at work | For this study, metrics were generated from administrative claims using the Medstat Market Scan HPM database. This database encompasses person-level information from 374,799 employees over a three-year period, specifically from 1997 to 1999. By utilizing this extensive database, we were able to gather valuable insights into the productivity challenges faced by individuals with type 2 diabetes.  In relation to the presenteeism, the number of unproductive days at work for individuals with T2D, with or without CVD, was extracted from a study by Goetzel et al. (14). On average, individuals with both T2D and CVD encountered 7 days of unproductive work time per year. | Goetzel et al. 2004 (14) |
| **13** | Dropout from work due to a non-fatal MI/stroke | When examining the impact of non-fatal MI on work absence, the study conducted by Worcester et al. in 2014 was the sole source providing relevant information. Their findings shed light on the number of days off from work attributed to non-fatal MI.  Additionally, Vyas et al., a Canadian study, reported the number of days off from work due to non-fatal stroke. With a study population of 91,633 respondents aged 18 to 70 years, we carefully reviewed the characteristics of the individuals included and found that this study aligned well with our own study population.  The model assumed that 9% of individuals who experienced an MI would not return to work. For those who experienced a stroke, the model assumed that 38% would not return to work. The average time to return to work after an MI was 60.34 days (15), and it was 61 days after a stroke (16). As specific data on early retirement for people with T2D were unavailable, the model assumed the early retirement rates to be similar to the general population of Qatar. | Days off from work due to non-fatal stroke from Vyas et al. 2016 (17).  Days off from work due to non-fatal MI from Worcester et al. 2014 (15).  The Ministry of Development Planning and Statistics (18).  The Worldometer database (19).  The Statista and Worldbank databases (20) |

| **14** | Average wages in Qatar for the working full-time population | The Ministry of Development Planning and Statistics in Qatar has introduced a groundbreaking initiative known as the National Development Strategy.This strategy serves as a comprehensive roadmap, guiding Qatar's progress in various domains including economic growth, social advancement, cultural enrichment, and environmental sustainability. By implementing this strategy, Qatar aims to foster holistic development and ensure a prosperous future for its people.  The Worldometer database: is a comprehensive global resource that provides valuable statistics across a wide range of areas. It covers essential aspects such as population, government and economics, society and media, environment, food, water, energy, and health. By drawing from this extensive database, researchers and analysts gain access to reliable and up-to-date information to support their studies and decision-making processes.  Statista is a prominent global platform for data and business intelligence. With a vast collection of statistics, reports, and insights from over 80,000 topics and 22,500 sources, it offers valuable insights into various industries. Researchers and professionals can utilize this platform to access in-depth information that aids in understanding market trends, consumer behavior, and industry performance. The World Bank database stands as one of the world's largest sources of funding and knowledge for developing countries. Committed to reducing poverty, promoting sustainable development, and fostering shared prosperity, the World Bank and its five institutions provide valuable data, research, and expertise. This database serves as a vital resource for economists, policymakers, and analysts seeking to gain a deeper understanding of global development trends and challenges. |  |
| --- | --- | --- | --- |
| **15** | The effect of type 2 diabetes on workforce | An article that provides information about the effect of diabetes on people productivity at work | Passey et al. 2012 (21) |

*ABS: Australian Bureau of Statistics, NDSS: The National Diabetes Services Scheme, CVD: cardiovascular disease, MI: myocardial infarction, NHS: National Health Survey, PCE-ASCVD: Pooled Cohort Equation-Atherosclerotic Cardiovascular Disease, AIHW: Australian Institute of Health and Welfare, REACH: global Atherothrombosis for Continued Health, HPM: Health and Productivity Management

**Supplementary Table 6.** **Description of the Interventional Cohorts and Data Sources Used in the Intervention Scenarios.**

**Supplementary Table 6.1**

| **Source** | **Description** |
| --- | --- |
| Cohort 1 (scenario 1) | The model incorporated a 9.5% reduction in the incidence of T2D, based on a population-based mathematical model conducted by Alareeki et al. in 2023 among individuals with T2D in Qatar (22). In the study, a deterministic population-based mathematical model was used to explore the impact of a composite health intervention on the epidemiology of T2D in the Qatari population. The composite intervention included weight reduction of ≥5%, moderate intensity physical activity of ≥30 min/day, dietary fat intake of <30% of total energy, saturated fat intake of <10%, and fiber intake of ≥15 g/1,000 kcal. The study spanned a three-decade time horizon, up to 2050, to capture the long-term impact of the interventions. The effect of the intervention was evaluated by comparing the predicted incidence and prevalence of T2D with and without the intervention. Of note, in our model, individuals who did not develop incident diabetes (accounting for 9.5% of the population) were replaced by healthy individuals. To account for the higher productivity observed in the healthy population, productivity indices were adjusted accordingly (14,23). |
| Cohort 2 (scenario 2) | The model implemented a 17% reduction in hypertension using the PCE-ASCVD algorithm, drawing from a meta-analysis of randomized clinical trials (RCTs) including 14,094 people conducted by Aggarwal et al. in 2018 (24). The findings of the study demonstrated the significant impact of intensive SBP lowering on reducing the incidence of CVD outcomes. To account for variations in SBP thresholds across countries and potential changes over time, our models utilized the SBP threshold of ≤130 mmHg based on guideline recommendations (25). The reduction in SBP was specifically applied to individuals whose initial levels exceeded the guideline-recommended threshold (>130/80 mmHg) (25). |
| Cohort 3 (scenario 3) | The model integrated a 19% reduction in the number of smokers using the PCE-ASCVD algorithm. This reduction was derived from a retrospective analysis conducted on prospectively collected data from the Framingham Heart Study, which involved 8,770 individuals without CVD (26). The study emphasized that quitting smoking among heavy smokers was associated with a significantly lower risk of developing CVD within a span of 5 years when compared to current smokers. |
| Cohort 4 (scenario 4) | The model hypothesized a 1 mmol/L decrease in total cholesterol via the PCE-ASCVD algorithm. This estimation was derived from a meta-analysis of 14 RCTs, involving 18,686 people with diabetes at high risk of vascular events (27). The study revealed a significant 21% proportional decrease in major vascular events, per 1 mmol/L reduction in LDL cholesterol, among individuals with diabetes receiving statins over a follow-up period of 4.3 years. |

**Supplementary Table 6.2**

| **Source** | **Description** |
| --- | --- |
| Alareeki et al. in 2023 (22) | To explore the effects of health interventions on the epidemiology of type 2 diabetes (T2D) among the population in Qatar, a deterministic population-based mathematical model was employed. This study focused on individuals aged 20 to 79 years and aimed to assess the impact of various interventions over a three-decade time horizon, up to the year 2050. By allowing for this extended timeframe, the study considered the long-term consequences of different intervention approaches.  The impact of each intervention was evaluated by comparing the predicted incidence and prevalence of T2D with the intervention in place against a counterfactual scenario without any intervention. This comparative analysis provided insights into the effectiveness of different intervention strategies and their potential for mitigating the burden of T2D in the population of Qatar. |
| Aggarwal et al. in 2018 (24) | This meta-analysis employed a comprehensive approach by pooling individual patient data from the SPRINT and ACCORD trials. The objective was to evaluate whether targeting intensive blood pressure levels (SBP <120 mmHg) or standard targets (SBP <140 mmHg) could effectively reduce the risk of cardiovascular disease (CVD) in individuals with diabetes.  The study findings demonstrated a significant reduction in composite outcomes, encompassing myocardial infarction, other acute coronary syndromes, stroke, heart failure, and cardiovascular mortality, with the implementation of intensive blood pressure management. The hazard ratio of 0.83 (95% CI 0.74-0.92, P<0.001) indicated a substantial reduction in the risk of these adverse events among the diabetic population. |
| Duncan et al. in 2019 (26) | A retrospective analysis was conducted on data collected from participants of the renowned Framingham Heart Study. This analysis included two cohorts: the original cohort (N=3805), who underwent their fourth examination between 1954 and 1958, and the offspring cohort (N=4965), who underwent their first examination between 1971 and 1975. The aim of the study was to investigate the relationship between the duration of smoking cessation and the occurrence of cardiovascular disease (CVD).  In comparison to individuals who were currently smoking, those who had quit smoking within the past five years exhibited significantly lower rates of incident CVD. The incidence rates per 1000 person-years were 11.56 (95% CI, 10.30-12.98) for current smokers and 6.94 (95% CI, 5.61-8.59) for individuals who had ceased smoking within five years. This yielded a difference in incidence rates of -4.51 (95% CI, -5.90 to -2.77). Moreover, the risk of developing incident CVD among those who quit smoking within five years was notably lower, with a hazard ratio of 0.61 (95% CI, 0.49-0.76). |
| Cholesterol Treatment Trialists’ Collaborators in 2008 (27) | The study examined data from a substantial cohort of 18,686 individuals with diabetes who were at a high risk of vascular events. Among these participants, 17,220 had type 2 diabetes. The analysis drew from 14 randomized trials involving statins, a class of medications commonly used to lower LDL cholesterol. By obtaining weighted estimates, the researchers assessed the impact of reducing LDL cholesterol levels by 1 mmol/L on various clinical outcomes.  During an average follow-up period of 4.3 years, the study investigated the effects of statins on the occurrence of clinical outcomes. |

*CVD: cardiovascular disease, SBP: systolic blood pressure, HDL: high-density lipoprotein cholesterol, CI: confidence interval, RCT: randomized controlled trial, AHEAD: the Action for Health in Diabetes, MI: myocardial infarction, SI: ischemic stroke.

**Supplementary Table 7. Model Input Variables and Distributions**

| Input variable | | Distribution | Point estimate | Standard error | Alpha | Beta | Uncertainty range |
| --- | --- | --- | --- | --- | --- | --- | --- |
| Proportions of events for type 2 diabetes at risk of CVD | | | | | | | |
| Non-fatal MI | | Beta | 0.83 | 0.002 | 25,558 | 5,235 | ±95% confidence interval |
| Fatal MI | | Beta | 0.17 | 0.003 | 2,068 | 10,096 |  |
| Non-fatal stroke | | Beta | 0.99 | 0.003 | 846 | 9 |  |
| Fatal stroke | | Beta | 0.83 | 0.002 | 25,558 | 5235 |  |
| Proportions of events for type 2 diabetes with established CVD population | | | | | | | |
| Non-fatal MI | | Beta | 0.83 | 0.002 | 25558 | 5235 | ±95% confidence interval |
| Non-fatal MI and stroke | | Beta | 0.17 | 0.003 | 2068 | 10096 |  |
| Non-fatal stroke | | Beta | 0.99 | 0.003 | 846 | 9 |  |
| Non-fatal stroke and MI | | Beta | 0.83 | 0.002 | 25558 | 5235 |  |
| Transition probabilities for recurrent events for type 2 diabetes | | | | | | | |
| CAD alone | CV death | Beta | 0.023 | 0.001 | 253 | 10,729 | ±95% confidence interval |
|  | Non-fatal MI | Beta | 0.018 | 0.001 | 198 | 10,784 |  |
|  | Non-fatal stroke | Beta | 0.016 | 0.001 | 176 | 10,806 |  |
| CVD alone | CV death | Beta | 0.021 | 0.002 | 80 | 3,728 |  |
|  | Non-fatal MI | Beta | 0.015 | 0.002 | 57 | 3,751 |  |
|  | Non-fatal stroke | Beta | 0.042 | 0.003 | 160 | 3,648 |  |
| CAD and CVD | CV death | Beta | 0.026 | 0.002 | 128 | 5,210 |  |
|  | Non-fatal MI | Beta | 0.017 | 0.002 | 92 | 5,246 |  |
|  | Non-fatal stroke | Beta | 0.035 | 0.003 | 189 | 5,149 |  |
| Productivity index | | | | | | | |
| Absenteeism | | | | | | | |
|  | Diabetes with MI | Beta | 0.23 | 0.10 | 5.30 | 0.043 | Assuming 10% standard error to the mean |
|  | Diabetes without MI | Beta | 0.81 | 0.10 | 65.41 | 0.012 |  |
|  | Diabetes with stroke | Beta | 0.25 | 0.10 | 6.27 | 0.040 |  |
|  | Diabetes without stroke | Beta | 0.81 | 0.10 | 65.74 | 0.012 |  |
|  | No diabetes and no MI | Beta | 0.98 | 0.10 | 96.06 | 0.010 |  |
|  | No diabetes no stroke | Beta | 0.98 | 0.10 | 96.06 | 0.010 |  |
| Presenteeism | | | | | | | |
|  | Diabetes with CVD | Beta | 0.97 | 0.10 | 94.09 | 0.010 | Assuming 10% standard error to the mean |
|  | Diabetes without CVD | Beta | 0.97 | 0.10 | 94.09 | 0.010 |  |
| Early exit from workforce | | | | | | | |
|  | Return to work after MI | Beta | 0.75 | 0.10 | 56.04 | 0.013 | Assuming 10% standard error to the mean |
|  | Return to work after stroke | Beta | 0.67 | 0.10 | 44.44 | 0.015 |  |
|  | Return to work after no MI | Beta | 1.00 | 0.10 | 100.00 | 0.010 |  |
|  | Return to work after no stroke | Beta | 1.00 | 0.10 | 100.00 | 0.010 |  |

*MI: myocardial infarction, CVD: cardiovascular disease, CAD: coronary artery disease.

**Supplementary Table 8. Impact on Years of Life Lived in a Qatari Working-Age Cohort with Type 2 Diabetes and First Cardiovascular Disease Events.**

1. **Men**

| **Year** | **2024** | **2025** | **2026** | **2027** | **2028** | **2029** | **2030** | **2031** | **2032** | **2033** | **Sum (2024-33)** |
| --- | --- | --- | --- | --- | --- | --- | --- | --- | --- | --- | --- |
| **Base-case** | 166,978 | 334,331 | 344,290 | 353,287 | 360,928 | 365,387 | 366,832 | 366,888 | 365,343 | 362,197 | 3,386,461 |
| **Cohort 1 (scenario 1)** | 667,881 | 688,650 | 707,753 | 724,514 | 736,655 | 742,523 | 743,961 | 742,373 | 737,545 | 362,535 | 6,854,391 |
| **Difference (cohorts base-case and 1)** | 500,903 | 354,319 | 363,463 | 371,227 | 375,727 | 377,136 | 377,129 | 375,485 | 372,202 | 338 | 3,467,930 |
| **Cohort 2 (scenario 2)** | 326,639 | 343,047 | 358,602 | 373,046 | 385,934 | 395,374 | 401,534 | 406,045 | 408,689 | 409,483 | 3,808,391 |
| **Difference (cohorts base-case and 2)** | 159,661 | 8,716 | 14,312 | 19,759 | 25,005 | 29,987 | 34,702 | 39,157 | 43,345 | 47,287 | 421,930 |
| **Cohort 3 (scenario 3)** | 326,563 | 342,820 | 358,226 | 372,520 | 385,261 | 394,560 | 400,586 | 404,969 | 407,492 | 408,171 | 3,801,168 |
| **Difference (cohorts base-case and 3)** | 159,585 | 8,489 | 13,936 | 19,233 | 24,333 | 29,173 | 33,754 | 38,081 | 42,148 | 45,974 | 414,707 |
| **Cohort 4 (scenario 4)** | 169,876 | 342,859 | 358,291 | 372,611 | 385,378 | 394,702 | 400,753 | 405,161 | 407,707 | 408,409 | 3,645,748 |
| **Difference (cohorts base-case and 4)** | 2,898 | 8,528 | 14,001 | 19,324 | 24,449 | 29,315 | 33,922 | 38,273 | 42,364 | 46,212 | 259,287 |

1. **Women**

| **Year** | **2024** | **2025** | **2026** | **2027** | **2028** | **2029** | **2030** | **2031** | **2032** | **2033** | **Sum (2024-33)** |
| --- | --- | --- | --- | --- | --- | --- | --- | --- | --- | --- | --- |
| **Base-case** | 45,887 | 92,596 | 96,627 | 100,199 | 103,286 | 105,575 | 107,198 | 108,534 | 109,509 | 110,070 | 979,480 |
| **Cohort 1 (scenario 1)** | 183,317 | 191,746 | 199,285 | 205,876 | 211,171 | 215,161 | 218,354 | 220,721 | 223,968 | 112,107 | 1,981,706 |
| **Difference (cohorts base-case and 1)** | 137,430 | 99,150 | 102,658 | 105,677 | 107,885 | 109,587 | 111,157 | 112,188 | 114,458 | 2,036 | 1,002,225 |
| **Cohort 2 (scenario 2)** | 88,192 | 92,762 | 96,905 | 100,589 | 103,789 | 106,187 | 107,917 | 109,359 | 110,440 | 111,105 | 1,027,246 |
| **Difference (cohorts base-case and 2)** | 42,305 | 166 | 278 | 391 | 503 | 613 | 719 | 826 | 931 | 1,035 | 47,766 |
| **Cohort 3 (scenario 3)** | 88,154 | 92,645 | 96,710 | 100,315 | 103,436 | 105,758 | 107,413 | 108,781 | 109,788 | 110,379 | 1,023,379 |
| **Difference (cohorts base-case and 3)** | 42,267 | 49 | 83 | 116 | 150 | 183 | 216 | 248 | 279 | 308 | 43,899 |
| **Cohort 4 (scenario 4)** | 45,892 | 92,607 | 96,644 | 100,325 | 103,386 | 105,738 | 107,423 | 108,820 | 109,856 | 110,476 | 981,167 |
| **Difference (cohorts base-case and 4)** | 5 | 11 | 17 | 126 | 100 | 163 | 225 | 287 | 347 | 406 | 1,687 |

*Base-case: original model with original estimates from the Primary Healthcare Corporation without any adjustment in risk factors.

Cohort 1 (scenario 1): the model assumed a 58% reduction in the incidence of type 2 diabetes.

Cohort 2 (scenario 2): the model assumed a 17% reduction in hypertension.

Cohort 3 (scenario 3): the model assumed a 19% reduction in the number of smokers.

Cohort 4 (scenario 4): the model assumed a 39 mg/dl reduction in hypercholesterolemia.

**Supplementary Table 9. Impact on Productivity-Adjusted Life Years (PALYs) in a Qatari Working-Age Cohort with Type 2 Diabetes and First Cardiovascular Disease Events.**

1. **Men**

| **Year** | **2024** | **2025** | **2026** | **2027** | **2028** | **2029** | **2030** | **2031** | **2032** | **2033** | **Sum (2024-33)** |
| --- | --- | --- | --- | --- | --- | --- | --- | --- | --- | --- | --- |
| **Base-case** | 72,596 | 145,356 | 149,685 | 153,597 | 156,919 | 158,858 | 159,486 | 159,510 | 158,839 | 157,471 | 1,472,316 |
| **Cohort 1 (scenario 1)** | 144,279 | 148,774 | 152,974 | 156,763 | 159,969 | 161,797 | 162,319 | 162,244 | 161,478 | 160,020 | 1,570,616 |
| **Difference (cohorts base-case and 1)** | 71,682 | 3,418 | 3,289 | 3,166 | 3,050 | 2,939 | 2,834 | 2,734 | 2,639 | 2,549 | 98,300 |
| **Cohort 2 (scenario 2)** | 142,011 | 149,145 | 155,908 | 162,187 | 167,791 | 171,895 | 174,573 | 176,534 | 177,684 | 178,029 | 1,655,756 |
| **Difference (cohorts base-case and 2)** | 69,415 | 3,789 | 6,223 | 8,590 | 10,871 | 13,037 | 15,087 | 17,024 | 18,845 | 20,559 | 183,440 |
| **Cohort 3 (scenario 3)** | 141,978 | 149,046 | 155,744 | 161,959 | 167,498 | 171,541 | 174,161 | 176,066 | 177,163 | 177,459 | 1,652,616 |
| **Difference (cohorts base-case and 3)** | 69,382 | 3,691 | 6,059 | 8,362 | 10,579 | 12,684 | 14,675 | 16,556 | 18,325 | 19,988 | 180,300 |
| **Cohort 4 (scenario 4)** | 73,856 | 149,063 | 155,773 | 161,998 | 167,549 | 171,603 | 174,234 | 176,150 | 177,257 | 177,562 | 1,585,045 |
| **Difference (cohorts base-case and 4)** | 1,260 | 3,708 | 6,087 | 8,401 | 10,630 | 12,745 | 14,748 | 16,640 | 18,418 | 20,092 | 112,729 |

1. **Women**

| **Year** | **2024** | **2025** | **2026** | **2027** | **2028** | **2029** | **2030** | **2031** | **2032** | **2033** | **Sum (2024-33)** |
| --- | --- | --- | --- | --- | --- | --- | --- | --- | --- | --- | --- |
| **Base-case** | 19,950 | 40,258 | 42,010 | 43,563 | 44,905 | 45,900 | 46,606 | 47,187 | 47,611 | 47,855 | 425,844 |
| **Cohort 1 (scenario 1)** | 39,826 | 41,680 | 43,351 | 44,827 | 46,095 | 47,019 | 47,729 | 48,313 | 48,673 | 49,624 | 457,137 |
| **Difference (cohorts base-case and 1)** | 19,876 | 1,422 | 1,341 | 1,264 | 1,190 | 1,119 | 1,123 | 1,127 | 1,062 | 1,769 | 31,293 |
| **Cohort 2 (scenario 2)** | 38,343 | 40,330 | 42,131 | 43,733 | 45,124 | 46,167 | 46,919 | 47,546 | 48,016 | 48,305 | 446,611 |
| **Difference (cohorts base-case and 2)** | 18,393 | 72 | 121 | 170 | 219 | 266 | 313 | 359 | 405 | 450 | 20,767 |
| **Cohort 3 (scenario 3)** | 38,326 | 40,279 | 42,046 | 43,613 | 44,970 | 45,980 | 46,700 | 47,294 | 47,732 | 47,989 | 444,930 |
| **Difference (cohorts base-case and 3)** | 18,376 | 21 | 36 | 51 | 65 | 80 | 94 | 108 | 121 | 134 | 19,086 |
| **Cohort 4 (scenario 4)** | 19,952 | 40,262 | 42,017 | 43,618 | 44,949 | 45,971 | 46,704 | 47,311 | 47,762 | 48,031 | 426,578 |
| **Difference (cohorts base-case and 4)** | 2 | 5 | 7 | 55 | 43 | 71 | 98 | 125 | 151 | 177 | 733 |

*Base-case: original model with original estimates from the Primary Healthcare Corporation without any adjustment in risk factors.

Cohort 1 (scenario 1): the model assumed a 58% reduction in the incidence of type 2 diabetes.

Cohort 2 (scenario 2): the model assumed a 17% reduction in hypertension.

Cohort 3 (scenario 3): the model assumed a 19% reduction in the number of smokers.

Cohort 4 (scenario 4): the model assumed a 39 mg/dl reduction in hypercholesterolemia.

**Supplementary Table 10. Impact on Cost of Productivity-Adjusted Life Years (PALYs) in a Qatari Working-Age Cohort with Type 2 Diabetes and First Cardiovascular Disease Events.**

**a. Men**

| **Year** | **2024** | **2025** | **2026** | **2027** | **2028** | **2029** | **2030** | **2031** | **2032** | **2033** | **Sum (2024-33)** |
| --- | --- | --- | --- | --- | --- | --- | --- | --- | --- | --- | --- |
| **Base-case** | $ 6,350,927,242 | $ 13,250,188,565 | $ 14,217,961,531 | $ 15,202,281,631 | $ 16,183,395,402 | $ 17,071,409,107 | $ 17,858,734,843 | $ 18,611,659,232 | $ 19,311,712,160 | $ 19,949,484,455 | $ 158,007,754,167 |
| **Cohort 1 (scenario 1)** | $ 12,621,891,624 | $ 13,561,769,612 | $ 14,530,352,459 | $ 15,515,655,672 | $ 16,497,920,166 | $ 17,387,242,870 | $ 18,176,057,686 | $ 18,930,673,733 | $ 19,632,608,717 | $ 20,272,427,951 | $ 167,126,600,490 |
| **Difference (cohorts base-case and 1)** | $ 6,270,964,382 | $ 311,581,047 | $ 312,390,928 | $ 313,374,041 | $ 314,524,763 | $ 315,833,763 | $ 317,322,843 | $ 319,014,501 | $ 320,896,557 | $ 322,943,496 | $ 9,118,846,322 |
| **Cohort 2 (scenario 2)** | $ 12,423,550,578 | $ 13,595,604,942 | $ 14,809,011,355 | $ 16,052,511,910 | $ 17,304,582,557 | $ 18,472,423,998 | $ 19,548,158,708 | $ 20,598,027,976 | $ 21,602,898,701 | $ 22,553,987,038 | $ 176,960,757,763 |
| **Difference (cohorts base-case and 2)** | $ 6,072,623,336 | $ 345,416,378 | $ 591,049,824 | $ 850,230,279 | $ 1,121,187,154 | $ 1,401,014,891 | $ 1,689,423,865 | $ 1,986,368,745 | $ 2,291,186,542 | $ 2,604,502,583 | $ 18,953,003,596 |
| **Cohort 3 (scenario 3)** | $ 12,420,656,035 | $ 13,586,640,567 | $ 14,793,477,387 | $ 16,029,890,993 | $ 17,274,434,194 | $ 18,434,425,329 | $ 19,502,015,526 | $ 20,543,455,382 | $ 21,539,621,068 | $ 22,481,707,063 | $ 176,606,323,544 |
| **Difference (cohorts base-case and 3)** | $ 6,069,728,793 | $ 336,452,002 | $ 575,515,857 | $ 827,609,362 | $ 1,091,038,792 | $ 1,363,016,222 | $ 1,643,280,682 | $ 1,931,796,151 | $ 2,227,908,908 | $ 2,532,222,608 | $ 18,598,569,377 |
| **Cohort 4 (scenario 4)** | $ 6,461,139,983 | $ 13,588,188,834 | $ 14,796,159,528 | $ 16,033,795,189 | $ 17,279,657,080 | $ 18,441,069,737 | $ 19,510,172,093 | $ 20,553,199,604 | $ 21,551,018,066 | $ 22,494,818,270 | $ 170,709,218,384 |
| **Difference (cohorts base-case and 4)** | $ 110,212,740 | $ 338,000,269 | $ 578,197,998 | $ 831,513,558 | $ 1,096,261,678 | $ 1,369,660,630 | $ 1,651,437,249 | $ 1,941,540,373 | $ 2,239,305,906 | $ 2,545,333,815 | $ 12,701,464,217 |

**b.Women**

| **Year** | **2024** | **2025** | **2026** | **2027** | **2028** | **2029** | **2030** | **2031** | **2032** | **2033** | **Sum (2024-33)** |
| --- | --- | --- | --- | --- | --- | --- | --- | --- | --- | --- | --- |
| **Base-case** | $ 1,745,288,055 | $ 3,669,758,695 | $ 3,990,359,940 | $ 4,311,642,075 | $ 4,631,160,376 | $ 4,932,601,553 | $ 5,218,779,123 | $ 5,505,742,056 | $ 5,788,562,739 | $ 6,062,585,408 | $ 45,856,480,020 |
| **Cohort 1 (scenario 1)** | $ 3,484,068,017 | $ 3,799,383,685 | $ 4,117,735,087 | $ 4,436,710,032 | $ 4,753,862,772 | $ 5,052,877,823 | $ 5,344,584,411 | $ 5,637,210,617 | $ 5,917,690,329 | $ 6,286,719,113 | $ 48,830,841,885 |
| **Difference (cohorts base-case and 1)** | $ 1,738,779,962 | $ 129,624,990 | $ 127,375,148 | $ 125,067,957 | $ 122,702,396 | $ 120,276,269 | $ 125,805,288 | $ 131,468,562 | $ 129,127,589 | $ 224,133,704 | $ 2,974,361,865 |
| **Cohort 2 (scenario 2)** | $ 3,354,352,341 | $ 3,676,323,407 | $ 4,001,824,727 | $ 4,328,447,461 | $ 4,653,726,746 | $ 4,961,225,602 | $ 5,253,802,266 | $ 5,547,626,954 | $ 5,837,776,224 | $ 6,119,579,457 | $ 47,734,685,185 |
| **Difference (cohorts base-case and 2)** | $ 1,609,064,286 | $ 6,564,712 | $ 11,464,788 | $ 16,805,386 | $ 22,566,370 | $ 28,624,048 | $ 35,023,144 | $ 41,884,898 | $ 49,213,485 | $ 56,994,049 | $ 1,878,205,165 |
| **Cohort 3 (scenario 3)** | $ 3,352,879,084 | $ 3,671,718,082 | $ 3,993,777,684 | $ 4,316,648,612 | $ 4,637,884,909 | $ 4,941,160,615 | $ 5,229,283,916 | $ 5,518,304,324 | $ 5,803,287,373 | $ 6,079,567,383 | $ 47,544,511,983 |
| **Difference (cohorts base-case and 3)** | $ 1,607,591,029 | $ 1,959,386 | $ 3,417,745 | $ 5,006,538 | $ 6,724,533 | $ 8,559,062 | $ 10,504,793 | $ 12,562,269 | $ 14,724,634 | $ 16,981,975 | $ 1,688,031,963 |
| **Cohort 4 (scenario 4)** | $ 1,745,468,518 | $ 3,670,201,531 | $ 3,991,044,862 | $ 4,317,078,715 | $ 4,635,642,272 | $ 4,940,234,772 | $ 5,229,747,710 | $ 5,520,279,234 | $ 5,806,900,727 | $ 6,084,946,504 | $ 45,941,544,843 |
| **Difference (cohorts base-case and 4)** | $ 180,463 | $ 442,835 | $ 684,923 | $ 5,436,640 | $ 4,481,896 | $ 7,633,218 | $ 10,968,587 | $ 14,537,178 | $ 18,337,987 | $ 22,361,096 | $ 85,064,823 |

*Base-case: original model with original estimates from the Primary Healthcare Corporation without any adjustment in risk factors.

Cohort 1 (scenario 1): the model assumed a 58% reduction in the incidence of type 2 diabetes.

Cohort 2 (scenario 2): the model assumed a 17% reduction in hypertension.

Cohort 3 (scenario 3): the model assumed a 19% reduction in the number of smokers.

Cohort 4 (scenario 4): the model assumed a 39 mg/dl reduction in hypercholesterolemia.

**Supplementary Table 11. Impact on Years of Life Lived in a Qatari Working-Age Cohort with Type 2 Diabetes and Recurrent Cardiovascular Disease Events.**

1. **Men**

| **Year** | **2024** | **2025** | **2026** | **2027** | **2028** | **2029** | **2030** | **2031** | **2032** | **2033** | **Sum (2024-33)** |
| --- | --- | --- | --- | --- | --- | --- | --- | --- | --- | --- | --- |
| **Base-case** | 20,113 | 26,741 | 33,223 | 39,460 | 45,387 | 50,983 | 56,224 | 61,129 | 65,690 | 33,425 | 432,375 |
| **Cohort 1 (scenario 1)** | 22,276 | 28,820 | 35,219 | 41,376 | 47,226 | 52,747 | 57,917 | 62,754 | 67,250 | 34,178 | 449,763 |
| **Difference (cohorts base-case and 1)** | 2,163 | 2,079 | 1,996 | 1,916 | 1,839 | 1,764 | 1,693 | 1,625 | 1,560 | 753 | 17,388 |
| **Cohort 2 (scenario 2)** | 19,685 | 26,066 | 32,327 | 38,371 | 44,125 | 49,570 | 54,683 | 59,479 | 63,948 | 32,546 | 420,801 |
| **Difference (cohorts base-case and 2)** | (428) | (675) | (895) | (1,089) | (1,262) | (1,413) | (1,541) | (1,650) | (1,742) | (879) | (11,575) |
| **Cohort 3 (scenario 3)** | 19,840 | 26,200 | 32,443 | 38,470 | 44,209 | 49,641 | 54,743 | 59,529 | 63,989 | 32,564 | 421,627 |
| **Difference (cohorts base-case and 3)** | (273) | (542) | (780) | (990) | (1,178) | (1,342) | (1,481) | (1,600) | (1,701) | (860) | (10,748) |
| **Cohort 4 (scenario 4)** | 13,044 | 19,527 | 25,962 | 32,255 | 38,311 | 44,065 | 49,498 | 54,586 | 59,349 | 63,777 | 400,375 |
| **Difference (cohorts base-case and 4)** | (7,069) | (7,214) | (7,260) | (7,205) | (7,077) | (6,918) | (6,726) | (6,543) | (6,341) | 30,352 | (32,001) |

1. **Women**

| **Year** | **2024** | **2025** | **2026** | **2027** | **2028** | **2029** | **2030** | **2031** | **2032** | **2033** | **Sum (2024-33)** |
| --- | --- | --- | --- | --- | --- | --- | --- | --- | --- | --- | --- |
| **Base-case** | 1,062 | 1,414 | 1,769 | 2,120 | 2,460 | 2,798 | 3,132 | 3,457 | 3,767 | 1,928 | 23,907 |
| **Cohort 1 (scenario 1)** | 1,923 | 2,248 | 2,577 | 2,903 | 3,218 | 3,532 | 3,845 | 4,149 | 36,865 | 33,736 | 94,997 |
| **Difference (cohorts base-case and 1)** | 860 | 834 | 808 | 783 | 758 | 734 | 713 | 691 | 33,098 | 31,809 | 71,089 |
| **Cohort 2 (scenario 2)** | 977 | 1,279 | 1,590 | 1,903 | 2,209 | 2,517 | 2,826 | 3,129 | 3,420 | 1,753 | 21,603 |
| **Difference (cohorts base-case and 2)** | (85) | (135) | (179) | (217) | (251) | (281) | (307) | (328) | (347) | (175) | (2,304) |
| **Cohort 3 (scenario 3)** | 1,062 | 1,416 | 1,774 | 2,125 | 2,464 | 2,801 | 3,135 | 3,460 | 3,769 | 1,929 | 23,935 |
| **Difference (cohorts base-case and 3)** | - | 2 | 5 | 4 | 4 | 3 | 3 | 3 | 2 | 1 | 28 |
| **Cohort 4 (scenario 4)** | 692 | 1,032 | 1,373 | 1,717 | 2,059 | 2,389 | 2,716 | 3,041 | 3,357 | 3,657 | 22,031 |
| **Difference (cohorts base-case and 4)** | (371) | (382) | (396) | (403) | (402) | (409) | (416) | (417) | (410) | 1,729 | (1,876) |

*Base-case: original model with original estimates from the Primary Healthcare Corporation without any adjustment in risk factors.

Cohort 1 (scenario 1): the model assumed a 58% reduction in the incidence of type 2 diabetes.

Cohort 2 (scenario 2): the model assumed a 17% reduction in hypertension.

Cohort 3 (scenario 3): the model assumed a 19% reduction in the number of smokers.

Cohort 4 (scenario 4): the model assumed a 39 mg/dl reduction in hypercholesterolemia.

#Numbers between brackets represent negative findings.

**Supplementary Table 12. Impact on Productivity-Adjusted Life Years (PALYs) in a Qatari Working-Age Cohort with Type 2 Diabetes and Recurrent Cardiovascular Disease Events.**

1. **Men**

| **Year** | **2024** | **2025** | **2026** | **2027** | **2028** | **2029** | **2030** | **2031** | **2032** | **2033** | **Sum (2024-33)** |
| --- | --- | --- | --- | --- | --- | --- | --- | --- | --- | --- | --- |
| **Base-case** | 8,744 | 11,626 | 14,444 | 17,156 | 19,733 | 22,166 | 24,444 | 26,577 | 28,560 | 14,532 | 187,982 |
| **Cohort 1 (scenario 1)** | 12,381 | 15,148 | 17,853 | 20,456 | 22,928 | 25,258 | 27,438 | 29,476 | 31,367 | 15,893 | 218,198 |
| **Difference (cohorts base-case and 1)** | 3,637 | 3,521 | 3,409 | 3,301 | 3,195 | 3,093 | 2,994 | 2,899 | 2,807 | 1,361 | 30,216 |
| **Cohort 2 (scenario 2)** | 8,558 | 11,333 | 14,055 | 16,682 | 19,184 | 21,551 | 23,774 | 25,860 | 27,802 | 14,150 | 182,949 |
| **Difference (cohorts base-case and 2)** | (186) | (294) | (389) | (473) | (549) | (614) | (670) | (717) | (757) | (382) | (5,032) |
| **Cohort 3 (scenario 3)** | 8,626 | 11,391 | 14,105 | 16,725 | 19,221 | 21,582 | 23,800 | 25,881 | 27,820 | 14,158 | 183,309 |
| **Difference (cohorts base-case and 3)** | (119) | (235) | (339) | (430) | (512) | (584) | (644) | (696) | (740) | (374) | (4,673) |
| **Cohort 4 (scenario 4)** | 8,744 | 11,626 | 14,444 | 17,156 | 19,733 | 22,166 | 24,444 | 26,577 | 28,560 | 14,532 | 187,982 |
| **Difference (cohorts base-case and 4)** | - | - | - | - | - | - | - | - | - | - | - |

1. **Women**

| **Year** | **2024** | **2025** | **2026** | **2027** | **2028** | **2029** | **2030** | **2031** | **2032** | **2033** | **Sum (2024-33)** |
| --- | --- | --- | --- | --- | --- | --- | --- | --- | --- | --- | --- |
| **Base-case** | 462 | 615 | 769 | 922 | 1,070 | 1,216 | 1,362 | 1,503 | 1,638 | 838 | 10,394 |
| **Cohort 1 (scenario 1)** | 1,989 | 2,097 | 2,208 | 2,318 | 2,424 | 2,530 | 2,638 | 2,742 | 16,938 | 15,109 | 50,993 |
| **Difference (cohorts base-case and 1)** | 1,527 | 1,482 | 1,439 | 1,396 | 1,354 | 1,314 | 1,276 | 1,238 | 15,301 | 14,271 | 40,599 |
| **Cohort 2 (scenario 2)** | 425 | 556 | 691 | 827 | 961 | 1,094 | 1,228 | 1,360 | 1,487 | 762 | 9,392 |
| **Difference (cohorts base-case and 2)** | (37) | (59) | (78) | (94) | (109) | (122) | (133) | (143) | (151) | (76) | (1,002) |
| **Cohort 3 (scenario 3)** | 462 | 616 | 771 | 924 | 1,071 | 1,218 | 1,363 | 1,504 | 1,639 | 839 | 10,406 |
| **Difference (cohorts base-case and 3)** | - | 1 | 2 | 2 | 2 | 1 | 1 | 1 | 1 | 0 | 12 |
| **Cohort 4 (scenario 4)** | 462 | 615 | 769 | 922 | 1,070 | 1,216 | 1,362 | 1,503 | 1,638 | 838 | 10,394 |
| **Difference (cohorts base-case and 4)** | - | - | - | - | - | - | - | - | - | - | - |

*Base-case: original model with original estimates from the Primary Healthcare Corporation without any adjustment in risk factors.

Cohort 1 (scenario 1): the model assumed a 58% reduction in the incidence of type 2 diabetes.

Cohort 2 (scenario 2): the model assumed a 17% reduction in hypertension.

Cohort 3 (scenario 3): the model assumed a 19% reduction in the number of smokers.

Cohort 4 (scenario 4): the model assumed a 39 mg/dl reduction in hypercholesterolemia.

#Numbers between brackets represent negative findings.

**Supplementary Table 13. Impact on Cost of Productivity-Adjusted Life Years (PALYs) in a Qatari Working-Age Cohort with Type 2 Diabetes and Recurrent Cardiovascular Disease Events.**

**a. Men**

| **Year** | **2024** | **2025** | **2026** | **2027** | **2028** | **2029** | **2030** | **2031** | **2032** | **2033** | **Sum (2024-33)** |
| --- | --- | --- | --- | --- | --- | --- | --- | --- | --- | --- | --- |
| **Base-case** | $ 764,977,079 | $ 1,059,806,347 | $ 1,371,975,933 | $ 1,698,001,104 | $ 2,035,082,014 | $ 2,382,009,373 | $ 2,737,195,084 | $ 3,101,000,063 | $ 3,472,327,046 | $ 1,841,002,834 | $ 20,463,376,877 |
| **Cohort 1 (scenario 1)** | $ 1,083,137,505 | $ 1,380,809,272 | $ 1,695,811,099 | $ 2,024,672,157 | $ 2,364,589,667 | $ 2,714,368,679 | $ 3,072,454,211 | $ 3,439,219,240 | $ 3,813,573,301 | $ 2,013,388,559 | $ 23,602,023,690 |
| **Difference (cohorts base-case and 1)** | $ 318,160,426 | $ 321,002,925 | $ 323,835,167 | $ 326,671,052 | $ 329,507,653 | $ 332,359,305 | $ 335,259,127 | $ 338,219,177 | $ 341,246,255 | $ 172,385,725 | $ 3,138,646,813 |
| **Cohort 2 (scenario 2)** | $ 748,713,183 | $ 1,033,038,207 | $ 1,335,008,435 | $ 1,651,136,526 | $ 1,978,478,290 | $ 2,315,973,882 | $ 2,662,179,276 | $ 3,017,299,735 | $ 3,380,237,717 | $ 1,792,607,466 | $ 19,914,672,716 |
| **Difference (cohorts base-case and 2)** | $ (16,263,897) | $ (26,768,140) | $ (36,967,498) | $ (46,864,578) | $ (56,603,724) | $ (66,035,491) | $ (75,015,808) | $ (83,700,328) | $ (92,089,329) | $ (48,395,368) | $ (548,704,161) |
| **Cohort 3 (scenario 3)** | $ 754,585,881 | $ 1,038,343,231 | $ 1,339,775,658 | $ 1,655,400,742 | $ 1,982,261,653 | $ 2,319,295,035 | $ 2,665,074,794 | $ 3,019,812,136 | $ 3,382,407,651 | $ 1,793,617,138 | $ 19,950,573,919 |
| **Difference (cohorts base-case and 3)** | $ (10,391,198) | $ (21,463,116) | $ (32,200,275) | $ (42,600,362) | $ (52,820,361) | $ (62,714,338) | $ (72,120,289) | $ (81,187,927) | $ (89,919,395) | $ (47,385,696) | $ (512,802,959) |
| **Cohort 4 (scenario 4)** | $ 764,977,079 | $ 1,059,806,347 | $ 1,371,975,933 | $ 1,698,001,104 | $ 2,035,082,014 | $ 2,382,009,373 | $ 2,737,195,084 | $ 3,101,000,063 | $ 3,472,327,046 | $ 1,841,002,834 | $ 20,463,376,877 |
| **Difference (cohorts base-case and 4)** | $ - | $ - | $ - | $ - | $ - | $ - | $ - | $ - | $ - | $ - | $ - |

**b.Women**

| **Year** | **2024** | **2025** | **2026** | **2027** | **2028** | **2029** | **2030** | **2031** | **2032** | **2033** | **Sum (2024-33)** |
| --- | --- | --- | --- | --- | --- | --- | --- | --- | --- | --- | --- |
| **Base-case** | $ 40,410,899 | $ 56,030,942 | $ 73,052,185 | $ 91,245,456 | $ 110,316,402 | $ 130,706,335 | $ 152,484,662 | $ 175,390,425 | $ 199,097,135 | $ 106,167,442 | $ 1,134,901,884 |
| **Cohort 1 (scenario 1)** | $ 174,037,429 | $ 191,167,051 | $ 209,696,607 | $ 229,397,539 | $ 249,976,839 | $ 271,934,185 | $ 295,342,527 | $ 319,882,976 | $ 2,059,345,412 | $ 1,914,158,742 | $ 5,914,939,308 |
| **Difference (cohorts base-case and 1)** | $ 133,626,530 | $ 135,136,110 | $ 136,644,422 | $ 138,152,083 | $ 139,660,437 | $ 141,227,851 | $ 142,857,865 | $ 144,492,551 | $ 1,860,248,277 | $ 1,807,991,300 | $ 4,780,037,425 |
| **Cohort 2 (scenario 2)** | $ 37,160,136 | $ 50,684,853 | $ 65,674,242 | $ 81,897,769 | $ 99,059,978 | $ 117,584,919 | $ 137,558,973 | $ 158,737,277 | $ 180,774,744 | $ 96,538,415 | $ 1,025,671,305 |
| **Difference (cohorts base-case and 2)** | $ (3,250,763) | $ (5,346,089) | $ (7,377,943) | $ (9,347,687) | $ (11,256,425) | $ (13,121,416) | $ (14,925,689) | $ (16,653,148) | $ (18,322,392) | $ (9,629,027) | $ (109,230,579) |
| **Cohort 3 (scenario 3)** | $ 40,410,899 | $ 56,124,270 | $ 73,239,776 | $ 91,428,732 | $ 110,488,075 | $ 130,864,652 | $ 152,632,183 | $ 175,528,838 | $ 199,226,678 | $ 106,230,236 | $ 1,136,174,338 |
| **Difference (cohorts base-case and 3)** | $ - | $ 93,328 | $ 187,590 | $ 183,275 | $ 171,673 | $ 158,318 | $ 147,521 | $ 138,413 | $ 129,542 | $ 62,794 | $ 1,272,454 |
| **Cohort 4 (scenario 4)** | $ 40,410,899 | $ 56,030,942 | $ 73,052,185 | $ 91,245,456 | $ 110,316,402 | $ 130,706,335 | $ 152,484,662 | $ 175,390,425 | $ 199,097,135 | $ 106,167,442 | $ 1,134,901,884 |
| **Difference (cohorts base-case and 4)** | $ - | $ - | $ - | $ - | $ - | $ - | $ - | $ - | $ - | $ - | $ - |

*Base-case: original model with original estimates from the Primary Healthcare Corporation without any adjustment in risk factors.

Cohort 1 (scenario 1): the model assumed a 58% reduction in the incidence of type 2 diabetes.

Cohort 2 (scenario 2): the model assumed a 17% reduction in hypertension.

Cohort 3 (scenario 3): the model assumed a 19% reduction in the number of smokers.

Cohort 4 (scenario 4): the model assumed a 39 mg/dl reduction in hypercholesterolemia.

#Numbers between brackets represent negative findings.

**Supplementary Table 14. Impact on Years of Life Lived in a Qatari Working-Age Cohort with Type 2 Diabetes and First and Recurrent Cardiovascular Disease Events.**

In the total population with T2D and both first and recurrent CVD, the base-case cohort is projected to accrue 234,040 years of life lived in 2024 (166,978 for men and 45,887 for women). In 2033, these estimates are projected to rise significantly to 507,619 years of life lived (362,197 for men and 110,070 for women). Over the next decade, the base-case model projects 4,822,224 years of life lived (95% CI, 4,773,109–5,010,628), with 3,818,836 for men and 1,003,387 for women.

In terms of interventions, intervention 1 expects the cohort to accrue 875,397 years of life in 2024, which is projected to decrease to 542,556 years by 2033, resulting in a total of 9,380,856 years over the ten-year period. This scenario projects a gain of 4,558,633 years of life lived (95%) for the total cohort. In contrast, intervention 2 forecasts an increase in years of life lived from 435,493 in 2024 to 554,887 in 2033, leading to a decade total of 5,278,040 years, with an additional gain of 455,817 years (9%). Similarly, intervention 3 projects 435,618 years of life lived in 2024, rising to 553,043 years in 2033, accumulating 5,270,109 years over the decade and a projected gain of 447,885 years (9%). In intervention 4, the cohort is expected to accrue 229,503 years of life lived in 2024, increasing to 586,319 years in 2033, resulting in a total of 5,049,321 years over the ten years, with a projected gain of 227,097 years (5%).

1. **Men**

| **Year** | **2024** | **2025** | **2026** | **2027** | **2028** | **2029** | **2030** | **2031** | **2032** | **2033** | **Sum (2024-33)** |
| --- | --- | --- | --- | --- | --- | --- | --- | --- | --- | --- | --- |
| **Base-case** | 187,091 | 361,072 | 377,512 | 392,747 | 406,316 | 416,370 | 423,056 | 428,017 | 431,034 | 395,621 | 3,818,836 |
| **Cohort 1 (scenario 1)** | 690,157 | 717,470 | 742,972 | 765,890 | 783,881 | 795,270 | 801,877 | 805,127 | 804,795 | 396,713 | 7,304,154 |
| **Difference (cohorts base-case and 1)** | 503,066 | 356,398 | 365,459 | 373,143 | 377,566 | 378,900 | 378,822 | 377,110 | 373,762 | 1,092 | 3,485,318 |
| **Cohort 2 (scenario 2)** | 346,324 | 369,112 | 390,930 | 411,417 | 430,058 | 444,943 | 456,217 | 465,524 | 472,637 | 442,029 | 4,229,191 |
| **Difference (cohorts base-case and 2)** | 159,233 | 8,040 | 13,417 | 18,669 | 23,743 | 28,573 | 33,161 | 37,507 | 41,603 | 46,408 | 410,355 |
| **Cohort 3 (scenario 3)** | 346,402 | 369,020 | 390,669 | 410,990 | 429,470 | 444,201 | 455,328 | 464,498 | 471,481 | 440,735 | 4,222,795 |
| **Difference (cohorts base-case and 3)** | 159,312 | 7,948 | 13,156 | 18,243 | 23,155 | 27,831 | 32,273 | 36,481 | 40,447 | 45,114 | 403,959 |
| **Cohort 4 (scenario 4)** | 182,920 | 362,386 | 384,253 | 404,866 | 423,688 | 438,768 | 450,252 | 459,747 | 467,056 | 472,186 | 4,046,122 |
| **Difference (cohorts base-case and 4)** | (4,171) | 1,314 | 6,741 | 12,119 | 17,373 | 22,397 | 27,196 | 31,730 | 36,022 | 76,565 | 227,286 |

1. **Women**

| **Year** | **2024** | **2025** | **2026** | **2027** | **2028** | **2029** | **2030** | **2031** | **2032** | **2033** | **Sum (2024-33)** |
| --- | --- | --- | --- | --- | --- | --- | --- | --- | --- | --- | --- |
| **Base-case** | 46,949 | 94,010 | 98,396 | 102,319 | 105,746 | 108,372 | 110,330 | 111,991 | 113,276 | 111,998 | 1,003,387 |
| **Cohort 1 (scenario 1)** | 185,240 | 193,994 | 201,862 | 208,779 | 214,389 | 218,693 | 222,199 | 224,870 | 260,833 | 145,843 | 2,076,702 |
| **Difference (cohorts base-case and 1)** | 138,290 | 99,984 | 103,466 | 106,460 | 108,643 | 110,321 | 111,869 | 112,879 | 147,557 | 33,845 | 1,073,315 |
| **Cohort 2 (scenario 2)** | 89,169 | 94,040 | 98,495 | 102,492 | 105,999 | 108,704 | 110,743 | 112,488 | 113,860 | 112,858 | 1,048,849 |
| **Difference (cohorts base-case and 2)** | 42,220 | 31 | 99 | 173 | 252 | 332 | 413 | 497 | 584 | 860 | 45,462 |
| **Cohort 3 (scenario 3)** | 89,216 | 94,062 | 98,483 | 102,440 | 105,900 | 108,559 | 110,549 | 112,241 | 113,557 | 112,307 | 1,047,314 |
| **Difference (cohorts base-case and 3)** | 42,267 | 52 | 87 | 121 | 154 | 187 | 219 | 250 | 281 | 309 | 43,926 |
| **Cohort 4 (scenario 4)** | 46,584 | 93,639 | 98,016 | 102,042 | 105,445 | 108,127 | 110,139 | 111,861 | 113,213 | 114,133 | 1,003,199 |
| **Difference (cohorts base-case and 4)** | (366) | (371) | (380) | (277) | (302) | (246) | (191) | (130) | (63) | 2,135 | (189) |

*Base-case: original model with original estimates from the Primary Healthcare Corporation without any adjustment in risk factors.

Cohort 1 (scenario 1): the model assumed a 58% reduction in the incidence of type 2 diabetes.

Cohort 2 (scenario 2): the model assumed a 17% reduction in hypertension.

Cohort 3 (scenario 3): the model assumed a 19% reduction in the number of smokers.

Cohort 4 (scenario 4): the model assumed a 39 mg/dl reduction in hypercholesterolemia.

#Numbers between brackets represent negative findings.

**Supplementary Table 15. Impact on Productivity-Adjusted Life Years (PALYs) in a Qatari Working-Age Cohort with Type 2 Diabetes and First and Recurrent Cardiovascular Disease Events.**

In the total population with T2D and both first and recurrent CVD, the base-case cohort is expected to accrue 101,753 PALYs in 2024, contributing approximately US$8.90 billion to the country's GDP. In 2033, this figure increases to 220,695 PALYs, with an associated GDP contribution of around US$27.96 billion. Over the entire period from 2024 to 2033, the base-case model estimates a total of 2,096,536 PALYs (95% CI: 1,689,272-2,182,939), contributing approximately US$225.46 billion (95% CI: 223.09-229.57) to the GDP. Men are expected to accrue 1,660,298 PALYs (equivalent to US$178.47 billion), while women are projected to accumulate 436,238 PALYs (equivalent to US$46.99 billion).

In intervention 1, the cohort is expected to accrue 198,475 PALYs in 2024, contributing about US$17.36 billion to GDP. In 2033, this number rises to 240,646 PALYs, translating to approximately US$30.49 billion in GDP. Over the decade, the total accrual is 2,296,944 PALYs, contributing US$245.47 billion to GDP, which reflects projected gains of 200,408 PALYs (10%) and economic benefits of around US$20.01 billion (9%). Similarly, intervention 2 anticipates 189,337 PALYs in 2024, contributing roughly US$16.56 billion to GDP. This number is expected to increase to 241,246 PALYs in 2033, with a GDP contribution of about US$30.56 billion. The total accrued over the decade is projected to be 2,294,709 PALYs, contributing approximately US$245.64 billion to GDP, resulting in a gain of 198,173 PALYs (9%) and economic gains of about US$20.17 billion (9%). For intervention 3, the model estimates 189,392 PALYs in 2024, contributing around US$16.57 billion to GDP. In 2033, the cohort is expected to reach 240,444 PALYs, with a GDP contribution of approximately US$30.46 billion. Over the ten-year duration, the total is projected at 2,291,261 PALYs, contributing about US$245.24 billion to GDP, with a projected gain of 194,725 PALYs (9%) and an economic gain of US$19.78 billion (9%). Lastly, in intervention 4, the cohort is expected to accrue 103,014 PALYs in 2024, contributing approximately US$9.01 billion to GDP. This figure rises to 240,963 PALYs in 2033, contributing around US$30.53 billion to GDP. Over the decade, the total accrual is projected at 2,209,998 PALYs, resulting in a GDP contribution of about US$238.25 billion. The projected gain for this cohort is 113,462 PALYs (5%) and economic gains of US$12.79 billion (6%).

**a. Men**

| **Year** | **2024** | **2025** | **2026** | **2027** | **2028** | **2029** | **2030** | **2031** | **2032** | **2033** | **Sum (2024-33)** |
| --- | --- | --- | --- | --- | --- | --- | --- | --- | --- | --- | --- |
| **Base-case** | 81,341 | 156,982 | 164,129 | 170,753 | 176,652 | 181,023 | 183,930 | 186,087 | 187,399 | 172,002 | 1,660,298 |
| **Cohort 1 (scenario 1)** | 156,660 | 163,921 | 170,827 | 177,220 | 182,897 | 187,055 | 189,758 | 191,720 | 192,845 | 175,912 | 1,788,814 |
| **Difference (cohorts base-case and 1)** | 75,319 | 6,939 | 6,698 | 6,467 | 6,245 | 6,032 | 5,828 | 5,633 | 5,446 | 3,910 | 128,516 |
| **Cohort 2 (scenario 2)** | 150,570 | 160,477 | 169,963 | 178,870 | 186,974 | 193,446 | 198,347 | 202,394 | 205,486 | 192,179 | 1,838,706 |
| **Difference (cohorts base-case and 2)** | 69,229 | 3,496 | 5,833 | 8,117 | 10,323 | 12,423 | 14,417 | 16,307 | 18,088 | 20,177 | 178,408 |
| **Cohort 3 (scenario 3)** | 150,604 | 160,437 | 169,849 | 178,684 | 186,719 | 193,123 | 197,961 | 201,948 | 204,983 | 191,616 | 1,835,925 |
| **Difference (cohorts base-case and 3)** | 69,263 | 3,455 | 5,720 | 7,931 | 10,067 | 12,100 | 14,031 | 15,861 | 17,585 | 19,614 | 175,627 |
| **Cohort 4 (scenario 4)** | 82,600 | 160,690 | 170,217 | 179,154 | 187,282 | 193,769 | 198,678 | 202,727 | 205,817 | 192,094 | 1,773,027 |
| **Difference (cohorts base-case and 4)** | 1,260 | 3,708 | 6,087 | 8,401 | 10,630 | 12,745 | 14,748 | 16,640 | 18,418 | 20,092 | 112,729 |

**b.Women**

| **Year** | **2024** | **2025** | **2026** | **2027** | **2028** | **2029** | **2030** | **2031** | **2032** | **2033** | **Sum (2024-3b. 3)** |
| --- | --- | --- | --- | --- | --- | --- | --- | --- | --- | --- | --- |
| **Base-case** | 20,412 | 40,872 | 42,779 | 44,485 | 45,975 | 47,116 | 47,968 | 48,690 | 49,248 | 48,693 | 436,238 |
| **Cohort 1 (scenario 1)** | 41,815 | 43,777 | 45,559 | 47,144 | 48,519 | 49,550 | 50,367 | 51,055 | 65,611 | 64,733 | 508,130 |
| **Difference (cohorts base-case and 1)** | 21,403 | 2,904 | 2,780 | 2,659 | 2,544 | 2,433 | 2,399 | 2,365 | 16,363 | 16,041 | 71,891 |
| **Cohort 2 (scenario 2)** | 38,768 | 40,886 | 42,822 | 44,560 | 46,084 | 47,261 | 48,147 | 48,906 | 49,503 | 49,067 | 456,003 |
| **Difference (cohorts base-case and 2)** | 18,356 | 13 | 43 | 75 | 110 | 144 | 179 | 216 | 254 | 374 | 19,765 |
| **Cohort 3 (scenario 3)** | 38,788 | 40,895 | 42,817 | 44,537 | 46,042 | 47,198 | 48,063 | 48,799 | 49,371 | 48,827 | 455,336 |
| **Difference (cohorts base-case and 3)** | 18,376 | 23 | 38 | 52 | 67 | 81 | 95 | 109 | 122 | 135 | 19,098 |
| **Cohort 4 (scenario 4)** | 20,414 | 40,877 | 42,786 | 44,540 | 46,018 | 47,188 | 48,065 | 48,814 | 49,399 | 48,869 | 436,972 |
| **Difference (cohorts base-case and 4)** | 2 | 5 | 7 | 55 | 43 | 71 | 98 | 125 | 151 | 177 | 733 |

*Base-case: original model with original estimates from the Primary Healthcare Corporation without any adjustment in risk factors.

Cohort 1 (scenario 1): the model assumed a 58% reduction in the incidence of type 2 diabetes.

Cohort 2 (scenario 2): the model assumed a 17% reduction in hypertension.

Cohort 3 (scenario 3): the model assumed a 19% reduction in the number of smokers.

Cohort 4 (scenario 4): the model assumed a 39 mg/dl reduction in hypercholesterolemia.

#Numbers between brackets represent negative findings.

**Supplementary Table 16. Impact on Productivity-Adjusted Life Years (PALYs) in a Qatari Working-Age Cohort with Type 2 Diabetes and First and Recurrent Cardiovascular Disease Events.**

1. **Men**

| **Year** | **2024** | **2025** | **2026** | **2027** | **2028** | **2029** | **2030** | **2031** | **2032** | **2033** | **Sum (2024-33)** |
| --- | --- | --- | --- | --- | --- | --- | --- | --- | --- | --- | --- |
| **Base-case** | $ 7,115,904,322 | $ 14,309,994,912 | $ 15,589,937,463 | $ 16,900,282,735 | $ 18,218,477,416 | $ 19,453,418,480 | $ 20,595,929,927 | $ 21,712,659,295 | $ 22,784,039,205 | $ 21,790,487,289 | $ 178,471,131,045 |
| **Cohort 1 (scenario 1)** | $ 13,705,029,130 | $ 14,942,578,884 | $ 16,226,163,558 | $ 17,540,327,829 | $ 18,862,509,833 | $ 20,101,611,549 | $ 21,248,511,897 | $ 22,369,892,973 | $ 23,446,182,018 | $ 22,285,816,509 | $ 190,728,624,180 |
| **Difference (cohorts base-case and 1)** | $ 6,589,124,808 | $ 632,583,972 | $ 636,226,095 | $ 640,045,094 | $ 644,032,417 | $ 648,193,069 | $ 652,581,970 | $ 657,233,678 | $ 662,142,813 | $ 495,329,220 | $ 12,257,493,135 |
| **Cohort 2 (scenario 2)** | $ 13,172,263,761 | $ 14,628,643,149 | $ 16,144,019,789 | $ 17,703,648,436 | $ 19,283,060,847 | $ 20,788,397,880 | $ 22,210,337,983 | $ 23,615,327,712 | $ 24,983,136,418 | $ 24,346,594,504 | $ 196,875,430,480 |
| **Difference (cohorts base-case and 2)** | $ 6,056,359,439 | $ 318,648,238 | $ 554,082,326 | $ 803,365,701 | $ 1,064,583,431 | $ 1,334,979,400 | $ 1,614,408,057 | $ 1,902,668,417 | $ 2,199,097,213 | $ 2,556,107,215 | $ 18,404,299,435 |
| **Cohort 3 (scenario 3)** | $ 13,175,241,917 | $ 14,624,983,798 | $ 16,133,253,045 | $ 17,685,291,734 | $ 19,256,695,847 | $ 20,753,720,364 | $ 22,167,090,320 | $ 23,563,267,519 | $ 24,922,028,718 | $ 24,275,324,201 | $ 196,556,897,463 |
| **Difference (cohorts base-case and 3)** | $ 6,059,337,595 | $ 314,988,886 | $ 543,315,582 | $ 785,008,999 | $ 1,038,218,431 | $ 1,300,301,884 | $ 1,571,160,393 | $ 1,850,608,223 | $ 2,137,989,513 | $ 2,484,836,912 | $ 18,085,766,418 |
| **Cohort 4 (scenario 4)** | $ 7,226,117,062 | $ 14,647,995,181 | $ 16,168,135,461 | $ 17,731,796,293 | $ 19,314,739,094 | $ 20,823,079,110 | $ 22,247,367,176 | $ 23,654,199,668 | $ 25,023,345,111 | $ 24,335,821,104 | $ 191,172,595,261 |
| **Difference (cohorts base-case and 4)** | $ 110,212,740 | $ 338,000,269 | $ 578,197,998 | $ 831,513,558 | $ 1,096,261,678 | $ 1,369,660,630 | $ 1,651,437,249 | $ 1,941,540,373 | $ 2,239,305,906 | $ 2,545,333,815 | $ 12,701,464,217 |

1. **Women**

| **Year** | **2024** | **2025** | **2026** | **2027** | **2028** | **2029** | **2030** | **2031** | **2032** | **2033** | **Sum (2024-33)** |
| --- | --- | --- | --- | --- | --- | --- | --- | --- | --- | --- | --- |
| **Base-case** | $ 1,785,698,953 | $ 3,725,789,637 | $ 4,063,412,125 | $ 4,402,887,531 | $ 4,741,476,778 | $ 5,063,307,888 | $ 5,371,263,785 | $ 5,681,132,481 | $ 5,987,659,875 | $ 6,168,752,851 | $ 46,991,381,903 |
| **Cohort 1 (scenario 1)** | $ 3,658,105,446 | $ 3,990,550,736 | $ 4,327,431,695 | $ 4,666,107,571 | $ 5,003,839,611 | $ 5,324,812,008 | $ 5,639,926,938 | $ 5,957,093,594 | $ 7,977,035,741 | $ 8,200,877,855 | $ 54,745,781,193 |
| **Difference (cohorts base-case and 1)** | $ 1,872,406,493 | $ 264,761,099 | $ 264,019,570 | $ 263,220,040 | $ 262,362,833 | $ 261,504,120 | $ 268,663,153 | $ 275,961,113 | $ 1,989,375,866 | $ 2,032,125,004 | $ 7,754,399,290 |
| **Cohort 2 (scenario 2)** | $ 3,391,512,477 | $ 3,727,008,260 | $ 4,067,498,969 | $ 4,410,345,230 | $ 4,752,786,723 | $ 5,078,810,520 | $ 5,391,361,240 | $ 5,706,364,231 | $ 6,018,550,968 | $ 6,216,117,872 | $ 48,760,356,490 |
| **Difference (cohorts base-case and 2)** | $ 1,605,813,523 | $ 1,218,623 | $ 4,086,845 | $ 7,457,699 | $ 11,309,945 | $ 15,502,632 | $ 20,097,455 | $ 25,231,750 | $ 30,891,093 | $ 47,365,021 | $ 1,768,974,586 |
| **Cohort 3 (scenario 3)** | $ 3,393,289,982 | $ 3,727,842,352 | $ 4,067,017,460 | $ 4,408,077,344 | $ 4,748,372,984 | $ 5,072,025,267 | $ 5,381,916,099 | $ 5,693,833,162 | $ 6,002,514,051 | $ 6,185,797,620 | $ 48,680,686,321 |
| **Difference (cohorts base-case and 3)** | $ 1,607,591,029 | $ 2,052,715 | $ 3,605,335 | $ 5,189,813 | $ 6,896,206 | $ 8,717,379 | $ 10,652,314 | $ 12,700,681 | $ 14,854,176 | $ 17,044,769 | $ 1,689,304,417 |
| **Cohort 4 (scenario 4)** | $ 1,785,879,416 | $ 3,726,232,472 | $ 4,064,097,047 | $ 4,408,324,171 | $ 4,745,958,674 | $ 5,070,941,106 | $ 5,382,232,372 | $ 5,695,669,659 | $ 6,005,997,862 | $ 6,191,113,946 | $ 47,076,446,727 |
| **Difference (cohorts base-case and 4)** | $ 180,463 | $ 442,835 | $ 684,923 | $ 5,436,640 | $ 4,481,896 | $ 7,633,218 | $ 10,968,587 | $ 14,537,178 | $ 18,337,987 | $ 22,361,096 | $ 85,064,823 |

*Base-case: original model with original estimates from the Primary Healthcare Corporation without any adjustment in risk factors.

Cohort 1 (scenario 1): the model assumed a 58% reduction in the incidence of type 2 diabetes.

Cohort 2 (scenario 2): the model assumed a 17% reduction in hypertension.

Cohort 3 (scenario 3): the model assumed a 19% reduction in the number of smokers.

Cohort 4 (scenario 4): the model assumed a 39 mg/dl reduction in hypercholesterolemia.

#Numbers between brackets represent negative findings.

**Supplementary Table 17.** **Assessment of the Validation Status of Health-Economic Decision Models**

17.1 The Assessment of the Validation Status of Health-Economic decision models (AdViSHE) tool

| **Question** |  |
| --- | --- |
| Part A: Validation of the conceptual model |  |
| A1/ Face validity testing (conceptual model): Have experts been asked to judge the appropriateness of the conceptual model? | ✓ (ZA and DB) |
| A2/ Cross validity testing (conceptual model): Has this model been compared to other conceptual models found in the literature or clinical textbooks? | ✓  Our results were compared to previous studies, as mentioned in the manuscript. |
| Part B: Input data validation |  |
| B1/ Face validity testing (input data): Have experts been asked to judge the appropriateness of the input data? | ✓ (ZA and DB) |
| B2/ Model fit testing: When input parameters are based on regression models, have statistical tests been performed? | N/A |
| Part C: Validation of the computerized model |  |
| C1/ External review: Has the computerized model been examined by modelling experts? | ✓  Our results were compared to previous studies, as mentioned in the manuscript. |
| C2/ Extreme value testing: Has the model been run for specific, extreme sets of parameter values in order to detect any coding errors? | ✓ |
| C3/ Testing of traces: Have patients been tracked through the model to determine whether its logic is correct? | ✓ |
| C4/ Unit testing: Have individual sub-modules of the computerized model been tested? | ✓ |
| Part D: Operational validation |  |
| D1/ Face validity testing (model outcomes): Have experts been asked to judge the appropriateness of the model outcomes? | ✓ (ZA and DB) |
| D2/ Cross validation testing (model outcomes): Have the model outcomes been compared to the outcomes of other models that address similar problems? | ✓ |
| D3/ Validation against outcomes using alternative input data: Have the mod | ✓ |
| D4/ Validation against empirical data: Have the model outcomes been compared to empirical data? | ✓ |
| Part E: Other validation techniques |  |
| E1/ Other validation techniques: Have any other validation techniques been performed? | We used TECH-VER, as reported below in section 17.2 |

17.2 TECHnical VERification (TECH-VER) tool.

| **Test description** | **Expected result** | **Result** |
| --- | --- | --- |
| Model input (pre-analysis) calculations | | |
| Does the technology (drug/device, etc.) acquisition cost increase with higher prices? | N/A | N/A |
| Does the probability of an event, derived from an OR/RR/HR and baseline probability, increase with higher OR/RR/HR? | N/A | N/A |
| Additional check not in TECH-VER: Do the survival model predictions in Excel match those obtained from R? | N/A | N/A |
| Event/state calculations | | |
| The sum of the number of individuals at each health state should add up to the cohort size | Yes | Yes. Calculated in the trace |
| Check if all probabilities and number of individuals in a state are greater than or equal to 0 | Yes | Yes. Calculated in the trace |
| Check if all probabilities are smaller than or equal to 1 | Yes | Yes. Observed in the trace |
| Are the number of dead individuals in the previous period smaller than the number of dead individuals in the subsequent period | N/A | N/A |
| In case of lifetime horizon, check if all individuals are dead at the end of the time horizon | N/A | N/A |
| Are the QALYs equal to the life years if the utilities are set to 1? | N/A | N/A |
| Are the QALYs equal to zero if the utilities are set to zero? | N/A | N/A |
| If state utilities are lower, are QALYs lower? | N/A | N/A |
| Are costs zero if all costs are set to zero? | N/A | N/A |
| If mortality risk is set to zero, do individuals die? | N/A | N/A |
| If mortality risk is set to 1, do all individuals die in the first cycle? | N/A | N/A |
| If all decision options have the same effectiveness, are life years and QALYs the same? | N/A | N/A |
| If all decision options have the same effectiveness and costs, are all results the same? | N/A | N/A |
| Is the number of individuals alive in the model, the same or lower as in the general population? | Yes | Yes. |
| Is the QALY at each cycle, the same or lower than the general population? | N/A |  |
| If the inflation rate is higher, are the costs which are based on a reference from previous years higher too? | N/A | N/A |
| Is the sum of all ingoing and outgoing transition probabilities in a state in a given cycle the same as the change in number of individuals? | Yes | Yes. Tested in the model |
| Are the number of individuals entering a tunnel state the same as the number of individuals leaving the tunnel state? | Yes | Yes. Tested in the trace |
| If the treatment acquisition cost is greater, are the costs greater? | N/A | N/A |
| Are the time conversions for probabilities conducted correctly? | N/A | We used annual cycle |
| Result calculations | | |
| Do the more effective decision options yield greater QALYs and life years? | Yes | Yes. Tested in different scenario analyses. |
| Do the more costly decision options yield greater treatment costs? | N/A | N/A |
| Are the total life years greater than the total QALYs? | Yes | Yes. As found in the results. |
| Are the undiscounted results greater than the discounted results? | Yes | Yes. Tested in the model. |
| Is the ratio of the undiscounted total QALYs to the undiscounted total life years within the max and min of the utility inputs? | Yes | Yes. Tested in the model |
| Subgroup analysis results: Do subgroups with better baseline health have better outcomes? | N/A | N/A |
| Do the disaggregated results sum to the total results? | N/A | N/A |
| Are the life years with half-cycle correction lower than the life years without? | N/A | N/A |
| Are the discounted results equal to undiscounted if the discount rate is set to zero? | Yes | Yes. Tested in the model. |
| If discount rates are higher, are the discounted results smaller? | Yes | Yes. As reported in the scenario analysis |
| Is the ratio of the total undiscounted treatment cost to the average duration of treatment similar to the treatment-related unit acquisition cost? | N/A | N/A |
| If the effect of the decision option is doubled, is the incremental effect approximately doubled? | Yes | Yes. Tested in the model |
| *Uncertainty analysis calculations* | | |
| Are all necessary parameters subject to uncertainty included in the OWSA? | N/A | N/A. OWSA was not conducted |
| Does the OWSA include any parameters associated with joint uncertainty? | N/A |  |
| Are the upper and lower bounds used in the one-way sensitivity analysis using confidence intervals based on the statistical distribution assumed for that parameter? | N/A |  |
| Are the resulting ICER, incremental costs/QALYs with upper and lower bound of a parameter plausible and in line with a priori expectations? | N/A |  |
| Do all parameters used in the sensitivity analysis have appropriate associated distributions – upper and lower bounds should surround the deterministic value (i.e. upper bound ≥ mean ≥ lower bound) | N/A |  |
| Standard error and not standard deviation used in sampling | N/A | N/A |
| Lognormal/gamma distribution for HRs and costs/resource use | N/A | N/A |
| Beta for utilities and proportions/probabilities | N/A | N/A |
| Dirichlet for multinomial | N/A | N/A |
| Multivariate normal for correlated inputs | N/A | N/A |
| Normal for other variables as long as samples do not violate the requirement to remain positive when appropriate | N/A | N/A |
| Check PSA output mean costs, QALYs, and ICER compared with the deterministic results. Is there a large discrepancy? | No | N/A |
| If you take new PSA runs from the Microsoft Excel model do you get similar results? | N/A | N/A |
| Is(are) the CEAC line(Overall validation) in line with the CE scatter plots and the efficient frontier? | N/A | N/A |
| Does the PSA cloud demonstrate an unexpected behaviour or have an unusual shape? | N/A | N/A |
| Is the sum of all CEAC lines equal to 1 for all WTP values? | N/A | N/A |
| Do the explored scenario analyses provide a balanced view on the structural uncertainty (i.e. not always looking at more optimistic scenarios)? | Yes | Yes |
| Are the scenario analysis results plausible and in line with a priori expectations? | Yes | Yes |
| Check the correlation between two PSA results (i.e. costs/QALYs under the SoC and costs/QALYs under the comparator). Should be very low (very high) if different (same) random streams are used for different arms | N/A | N/A |
| If a certain seed is used for random number generation (or previously generated random numbers are used), check if they are scattered evenly between 0 and 1 when they are plotted | N/A | N/A |
| Is the mean of the parameter samples generated by the model similar to the point estimate for that parameter? Use graphical methods to examine distributions, functions | N/A | N/A |
| Do sensitivity analyses include any parameters associated with methodological/structural uncertainty? | Yes | Yes, as reported in the manuscript. |
| Value of information analysis if applicable: Was this implemented correctly? | N/A | N/A |
| Which types of analysis? | N/A | N/A |
| Is EVPI larger than all individual EVPPIs? | N/A | N/A |
| Is EVPPI for a (group of) parameters larger than the EVSI of that (group) of parameter(s)? | N/A |  |
| Are the results from EVPPI in line with OWSA or other parameter importance analysis (e.g. ANCOVA)? | N/A |  |
| Did the electronic model pass the black-box tests of the previous verification stages in all PSA iterations and in all scenario analysis settings? | N/A | N/A |
| Check if all sampled input parameters in the PSA are correctly linked to the corresponding event/state calculations | N/A | N/A |
| Overall validation/other supplementary checks | | |
| Compare the model outcomes with clinical inputs used in the model, findings from the literature, clinical expert knowledge and other model outcomes | Yes | The results were compared with local and international sources. |

*Advishe: Assessment of the Validation Status of Health-Economic decision models, CEAC: Cost-Effectiveness Acceptability Curve, CPRD: Clinical Practice Research Datalink, CTTC: Cholesterol Treatment Trialists’ Collaboration. CV: Cardiovascular. EVPI: Expected Value of Perfect Information, EVPPI: Expected Value of Perfect Parameter Information, EVSI: Expected Value of Sample Information, HR: Hazard Ratio, ICER: Incremental Cost-Effectiveness Ratio, LLT: Lipid Lowering Treatment, OR: Odds Ratio, OWSA: One-Way Sensitivity Analysis, PSA: Probabilistic Sensitivity Analysis. QALYs: Quality-Adjusted Life Years, RR: Risk Ratio. SoC: Standard of Care, WTP: Willingness To Pay.

**References**

1. Institute for Health Metrics and Evaluation. [Internet]. [cited 2020 Sep 30]. Available from: http://www.healthdata.org/about.

2. Awad SF, O’Flaherty M, El-Nahas KG, Al-Hamaq AO, Critchley JA, Abu-Raddad LJ. Preventing type 2 diabetes mellitus in Qatar by reducing obesity, smoking, and physical inactivity: mathematical modeling analyses. Popul Health Metr. 2019 Dec;17(1):20.

3. Bener A, Al-Hamaq AOAA. Predictions Burden of Diabetes and Economics Cost: Contributing Risk Factors of Changing Disease Prevalence and its Pandemic Impact to Qatar. Exp Clin Endocrinol diabetes Off journal, Ger Soc Endocrinol [and] Ger Diabetes Assoc. 2016 Sep;124(8):504–11.

4. Syed MA, Alnuaimi AS, Zainel AJ, A/Qotba HA. Prevalence of non-communicable diseases by age, gender and nationality in publicly funded primary care settings in Qatar. BMJ Nutr Prev Heal. 2019;2(1):20–9.

5. Goff DCJ, Lloyd-Jones DM, Bennett G, Coady S, D’Agostino RB, Gibbons R, et al. 2013 ACC/AHA guideline on the assessment of cardiovascular risk: a report of the American College of Cardiology/American Heart Association Task Force on Practice Guidelines. Circulation. 2014 Jun;129(25 Suppl 2):S49-73.

6. The Primary Health Care Corporation [Internet]. Available from: https://www.phcc.gov.qa/

7. El-Menyar AA, Albinali HA, Bener A, Mohammed I, Al Suwaidi J. Prevalence and impact of diabetes mellitus in patients with acute myocardial infarction: a 10-year experience. Angiology. 2009;60(6):683–8.

8. Khan FY, Yasin M, Abu-Khattab M, El Hiday AH, Errayes M, Lotf AK, et al. Stroke in Qatar: a first prospective hospital-based study of acute stroke. J stroke Cerebrovasc Dis Off J Natl Stroke Assoc. 2008;17(2):69–78.

9. Qatar Net Migration Rate 1950-2023 [Internet]. 2023. Available from: https://www.macrotrends.net/countries/QAT/qatar/net-migration#:~:text=The net migration rate for,a 8.94%25 decline from 2019.

10. Qatar QA: UCB Projection: Mortality Rate: Under 5 per 1000 Births [Internet]. Available from: https://www.ceicdata.com/en/qatar/demographic-projection/qa-ucb-projection-mortality-rate-under-5-per-1000-births

11. Krempf M, Parhofer KG, Steg PG, Bhatt DL, Ohman EM, Röther J, et al. Cardiovascular event rates in diabetic and nondiabetic individuals with and without established atherothrombosis (from the REduction of Atherothrombosis for Continued Health [REACH] Registry). Am J Cardiol. 2010 Mar;105(5):667–71.

12. Sørensen J, Ploug UJ. The Cost of Diabetes-Related Complications: Registry-Based Analysis of Days Absent from Work. Williams CC, editor. Econ Res Int [Internet]. 2013;2013:618039. Available from: https://doi.org/10.1155/2013/618039

13. Breton M-C, Guénette L, Amiche MA, Kayibanda J-F, Grégoire J-P, Moisan J. Burden of diabetes on the ability to work: a systematic review. Diabetes Care. 2013 Mar;36(3):740–9.

14. Goetzel RZ, Long SR, Ozminkowski RJ, Hawkins K, Wang S, Lynch W. Health, absence, disability, and presenteeism cost estimates of certain physical and mental health conditions affecting U.S. employers. J Occup Environ Med. 2004 Apr;46(4):398–412.

15. Worcester MU, Elliott PC, Turner A, Pereira JJ, Murphy BM, Le Grande MR, et al. Resumption of work after acute coronary syndrome or coronary artery bypass graft surgery. Heart Lung Circ. 2014 May;23(5):444–53.

16. Hackett ML, Glozier N, Jan S, Lindley R. Returning to paid employment after stroke: the Psychosocial Outcomes In StrokE (POISE) cohort study. PLoS One. 2012;7(7):e41795.

17. Vyas M V, Hackam DG, Silver FL, Laporte A, Kapral MK. Lost Productivity in Stroke Survivors: An Econometrics Analysis. Neuroepidemiology. 2016;47(3–4):164–70.

18. Ministry of Development Planning and Statistics. Births & Deaths In the State of Qatar, 2016. [Accessed 2020 April]. Available from: https://www.psa.gov.qa/en/statistics/Statistical%20Releases/Population/BirthsDeaths/2016/Birth_death_2016_EN.pdf.

19. 38- Qatar population (2021) live — Countrymeters [Internet]. Countrymeters.info. 2021 [cited 14 September 2021]. Available from: https://countrymeters.info/en/Qatar.

20. Qatar: Gross domestic product (GDP) per capita in current prices from 1989 to 2029. Available at: https://www.statista.com/statistics/379994/gross-domestic-product-gdp-per-capita-in-qatar/.

21. Passey ME, Shrestha RN, Bertram MY, Schofield DJ, Vos T, Callander EJ, et al. The impact of diabetes prevention on labour force participation and income of older Australians: an economic study. BMC Public Health [Internet]. 2012;12(1):16. Available from: https://doi.org/10.1186/1471-2458-12-16

22. Alareeki A, Awad SF, Critchley JA, El-Nahas KG, Al-Hamaq AO, Alyafei SA, et al. Epidemiological impact of public health interventions against diabetes in Qatar: mathematical modeling analyses. Front public Heal. 2023;11:1167807.

23. Persson S, Johansen P, Andersson E, Lindgren P, Thielke D, Thorsted BL, et al. Days absent from work as a result of complications associated with type 2 diabetes: Evidence from 20 years of linked national registry data in Sweden. Diabetes Obes Metab. 2020 Sep;22(9):1586–97.

24. Aggarwal R, Steinkamp J, Chiu N, Petrie B, Mirzan H. Intensive Blood Pressure Targets for Diabetic and Other High-Risk Populations: A Pooled Individual Patient Data Analysis. Hypertens (Dallas, Tex 1979). 2018 May;71(5):833–9.

25. 10. Cardiovascular Disease and Risk Management: Standards of Care in Diabetes—2024. Diabetes Care [Internet]. 2023 Dec 11;47(Supplement_1):S179–218. Available from: https://doi.org/10.2337/dc24-S010

26. Duncan MS, Freiberg MS, Greevy RAJ, Kundu S, Vasan RS, Tindle HA. Association of Smoking Cessation With Subsequent Risk of Cardiovascular Disease. JAMA. 2019 Aug;322(7):642–50.

27. Kearney PM, Blackwell L, Collins R, Keech A, Simes J, Peto R, et al. Efficacy of cholesterol-lowering therapy in 18,686 people with diabetes in 14 randomised trials of statins: a meta-analysis. Lancet (London, England). 2008 Jan;371(9607):117–25.
